# Small-Molecule Hormones: Molecular Mechanisms of Action

**DOI:** 10.1155/2013/601246

**Published:** 2013-02-28

**Authors:** Monika Puzianowska-Kuznicka, Eliza Pawlik-Pachucka, Magdalena Owczarz, Monika Budzińska, Jacek Polosak

**Affiliations:** ^1^Department of Human Epigenetics, Mossakowski Medical Research Centre, 5 Pawinskiego Street, 02-106 Warsaw, Poland; ^2^Department of Geriatrics and Gerontology, Medical Center of Postgraduate Education, 61/63 Kleczewska Street, 01-826 Warsaw, Poland

## Abstract

Small-molecule hormones play crucial roles in the development and in the maintenance of an adult mammalian organism. On the molecular level, they regulate a plethora of biological pathways. Part of their actions depends on their transcription-regulating properties, exerted by highly specific nuclear receptors which are hormone-dependent transcription factors. Nuclear hormone receptors interact with coactivators, corepressors, basal transcription factors, and other transcription factors in order to modulate the activity of target genes in a manner that is dependent on tissue, age and developmental and pathophysiological states. The biological effect of this mechanism becomes apparent not earlier than 30–60 minutes after hormonal stimulus. In addition, small-molecule hormones modify the function of the cell by a number of nongenomic mechanisms, involving interaction with proteins localized in the plasma membrane, in the cytoplasm, as well as with proteins localized in other cellular membranes and in nonnuclear cellular compartments. The identity of such proteins is still under investigation; however, it seems that extranuclear fractions of nuclear hormone receptors commonly serve this function. A direct interaction of small-molecule hormones with membrane phospholipids and with mRNA is also postulated. In these mechanisms, the reaction to hormonal stimulus appears within seconds or minutes.

## 1. Introduction

Molecular mechanisms of action of small-molecule hormones have been studied for decades. The biological function of these hormones was initially attributed mostly to their extranuclear activities presently referred to as nongenomic; however, the exact mechanisms of such actions were then not known. Subsequently, the majority of efforts were directed towards the clarification of the transcription-modifying function of these hormones bound to their nuclear receptors that are hormone-regulated transcription factors. This generated an enormous amount of information regarding the genomic action of hormones, the identity of their target genes, and so forth. It finally became apparent that the genomic action of hormones is insufficient to fully explain their biological roles, so that the nongenomic mechanisms are again being intensively studied. In this comprehensive paper we present basic information regarding the genomic and nongenomic mechanisms of action of small-molecule hormones, emphasizing the intermediary role of various proteins between the hormonal stimulus and the biological response of the cell. It should be noted, though, that although our current knowledge of the molecular mechanisms of action of these hormones is impressive, not all has been solved and many mechanisms still await explanation.

## 2. The Genomic Mechanism of Action of Small-Molecule Hormones

“Genomic mechanism of hormone action” refers to the regulation of target gene activity by hormones *via *their protein receptors, which also possess all the features of a transcription factor. This mechanism engages transcription and translation, and its biological effects are executed by a newly synthesized proteins. The first effects of engagement of this mechanism might be detected 30–60 minutes after its initiation; however, maximal effects are usually observed after several hours.

### 2.1. Nuclear Hormone Receptors

Nuclear receptors of small-molecule hormones belong to the superfamily of nuclear receptors, consisting of receptors for steroid hormones, thyroid hormone, vitamin D, retinoic acid and its derivatives, fatty acids, prostaglandins, and cholesterol derivatives, as well as of “orphan” receptors with unknown ligands. Small fractions of some of these receptors also act outside of the nucleus, in mechanisms generally called “nongenomic”, which are mediated by processes other than a direct binding of the receptor to DNA.

**Figure 1:**
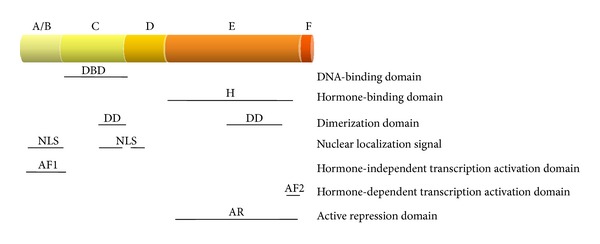
Schematic diagram of nuclear hormone receptor structure.

Structural similarities of nuclear receptors allow the subdivision of the superfamily into 7 families/subfamilies (0–VI); families I to VI are quite well defined [56–58], while family 0 contains various receptors, which do not fit into other families (Table 1). Nuclear receptors, although recognizing their own target genes and ligands with high specificity and being either partly or completely devoid of affinity for other genes and ligands, have a similar structure (Figure 1). A typical, full-length nuclear receptor has a variable A/B domain at its N-terminus, followed by a well-conserved DNA-binding C domain, then by a hinge D domain, and by a well-conserved ligand-binding E domain. Some receptors also have an F domain on their C-termini, the function of which is usually unclear.

The A/B domain of many nuclear receptors contains elements involved in hormone-independent transcription activation (AF1). Its function might be modified by phosphorylation, as was shown for the all-*trans*-retinoic acid receptor (RAR), peroxisome-proliferator-activated receptor (PPAR), orphan Nurr1 receptor, estrogen receptor (ER), and so forth [59–62]. The sequence and tridimensional structure of the C domain determine the recognition specificity of the receptor's target genes. The domain contains two zinc fingers; in each of them four perfectly conserved cysteines keep one zinc ion in place [63]. At the base of the first zinc finger, a P-box is present; its amino acid sequence determines the recognition of a specific (usually hexameric) DNA sequence in the receptor's target genes. At the base of the second zinc finger, a D-box is located; its sequence is, in turn, responsible for the recognition of the distance between the two hexamers forming the hormone response element (HRE) in the promoter of target gene [64]. In addition, the D-box plays a role in receptor dimerization. The C domain might contain the nuclear localization signal (NLS) or fragments thereof. Next, the D domain contains NLS and facilitates rotation of the DNA-binding domain in relation to the ligand-binding domain. In addition, it contains elements involved in cofactor binding, DNA binding, and in heterodimerization [65]. Finally, the E domain binds a specific hormone, takes part in homodimerization as well as in heterodimerization, and, on its C-terminal end, contains a ligand-dependent transcription activation domain (AF2) [66]. In some cases the E domain might play a role in the active inhibition of transcription. The E domain of the steroid hormone receptors takes part in the binding of heat shock proteins (HSP, chaperone). The structure of this domain is formed by 12 *α*-helices (H1–H12) and resembles pocket-like hormone-binding site. The sizes, shapes, and charges of this pocket present in various receptors differ from each other, and this why most receptors bind only their own hormones with an extremely high specificity and affinity; however, some of them, such as the PPAR*γ* receptor, possesses a large pocket allowing them to bind various ligands [67]. A very important feature of nuclear receptors is that in the absence of the hormone, conformation of their E domains differs from that acquired upon hormone binding [68–70]. The most spectacular is the change of position of the last helix (H12), containing the AF2 domain. Without the hormone, the H12 is moved to the side and protrudes from the rest of the E domain, leaving the empty pocket opened. Upon hormone binding, the H12 comes nearer and closes the hormone inside the pocket [71]. This feature is crucial for the majority of the functions of nuclear hormone receptors, including subcellular localization (as for steroid receptors) and transactivation activity.

The activity of the nuclear receptor might be modulated by various posttranscriptional modifications including phosphorylation, acetylation, methylation, palmitoylation, and sumoylation [72–76]. In addition, its biological efficiency depends on the rate of its turnover [77]. Like many other proteins, hormone receptors are degraded mainly by the ubiquitin-proteasome-dependent pathway. To be degraded by the proteasome, proteins must be tagged with multiple ubiquitins. The process of tagging depends on three enzymes acting sequentially; the third one, ubiquitin ligase, determines the specificity of protein ubiquitylation [78]; for example, Hdm2 and carboxyl-terminal HSP70 interacting protein (CHIP) promote degradation of the glucocorticoid receptor (GR) [79, 80]. Blocking receptor degradation by proteasome inhibitors impairs ER*α*- and progesterone-receptor-(PR-) mediated transactivation but enhances GR-mediated transactivation [81, 82]. Notably, binding of chaperones such as HSPs and associated proteins to steroid hormone receptor prevents receptor ubiquitylation [83, 84]. Calmodulin (CaM) binding to ER*α* also prevents receptor ubiquitylation and degradation by the proteasome [85, 86], while its binding to AR prevents receptor degradation by calpain [87]. In addition, palmitoylation of ER*α* decreases 17*β*-estradiol-dependent receptor degradation [88]. 

### 2.2. Hormone Response Elements in the Promoters of Target Genes

A classic, genomic mechanism of action of small-molecule hormones is based on the binding of its nuclear receptor to the target gene. Two elements facilitate such an interaction: the DNA-binding domain of the receptor and HRE, a specific sequence in the regulatory elements of the gene. Such sequences (single or multiple) are usually localized close to the basal promoter, not farther than several hundred base pairs in the 5′ direction from the transcription start site (TSS). However, they might also be present in atypical positions, for example, in the enhancers localized even a few thousand base pairs above the TSS. The negative HREs (nHREs) tend to localize close to TSS, sometimes even below this site [89, 90]. 

Analysis of the natural and artificial HREs showed that nuclear hormone receptors preferentially recognize hexamers, sequences consisting of six nucleotides. Steroid hormone nuclear receptors (family III), with the exception of ER, preferentially bind to the AGAACA sequence, while the remaining receptors, including families I and II receptors and ER, prefer the G/AGGTC/GA sequence [91–93]. Both are consensus sequences and consist of the nucleotides most commonly found at a given position in natural HREs; it is then to be expected that natural HREs very commonly differ from the consensus sequence. HREs usually are formed by two hexamers and, most commonly, nuclear hormone receptors bind to the DNA either as homodimers (mostly, but not exclusively, family III receptors) or as heterodimers (mostly families I and II receptors) [94–99]. The binding of a monomeric receptor to a monomeric or to a dimeric HRE is plausible, as in the case of steroidogenic factor-1 (SF-1, family V) [100], but for “classic” receptors such situations are less common. Depending on the relative position of the two hexamers, dimeric HRE might be a direct repeat (DR), palindromic (PAL), or inverted palindrome (IP) HRE. 

HREs for steroid hormone receptors, also called steroid hormone response elements (SREs), are usually palindromes consisting of the AGAACAnnnTGTTCT or of a similar sequences with three neutral (e.g., of any sequence) nucleotides between hexamers. As mentioned above, the exception to this rule is ER which preferentially binds to the G/AGGTC/GAnnnTC/GACCT/C palindrome [64, 101]. Nevertheless, each of these receptors preferentially recognizes its own target SREs with a very high specificity being a result of various factors, such as deviations from the SRE consensus sequence, distinct amino acids surrounding DNA binding domain fragments of the receptor directly contacting SRE, interactions with other transcription factors bound to their own binding sites in the proximity of SRE, tissue-specific expression of various receptor isoforms, and the level of receptor expression [102, 103]. It should be mentioned that other types of SREs are known, such as a selective androgen response element (ARE) which is not PAL, but DR-type. It has been recently shown that such AREs might be recognized not only by AR, but also by PR [104, 105]. In addition to classic SREs, which mediate transcription activation, a number of negative SREs are known that inhibit the transcription when the steroid-hormone-activated receptor binds to nSRE [106, 107]. 

Nuclear receptors belonging to the families I and II preferentially bind to the consensus G/AGGTC/GA sequence organized into DR, PAL, or IP [108–111]. The binding to DR drives the strongest biological effect; in fact, natural HREs recognized by these receptors are most commonly DRs. Specificity of the binding is achieved thanks to HRE's configuration, to the number of neutral nucleotides separating the two hexamers, to the sequence of hexamers and of HRE-flanking DNA-fragments, and to the sequence of the receptor DNA-binding domain [112–115]. In DRs, one neutral nucleotide between hexamers (DR1) warrants the binding of RXR/RXR homodimers, of RAR/RXR or of PPAR/RXR heterodimers; two nucleotides (DR2)—the binding of RAR/RXR heterodimer; three nucleotides (DR3)—the binding of VDR/RXR heterodimer; four nucleotides (DR4)—the binding of TR/RXR heterodimer; finally, five nucleotides (DR5) – the binding of RAR/RXR heterodimer. Nuclear receptors for nonsteroid small-molecule hormones also bind to DR0 and to DRs with more than five neutral nucleotides separating hexamers [116, 117] as well as to other nonclassical HREs. In addition, some HREs might be bound by various receptors; for example, the AGGTCATGACCT PAL0 sequence is recognized by TR-, VDR-, and RAR-containing dimers [118–120]. Specific nHREs are also known for practically all nuclear hormone receptors belonging to the families I and II [121–124].

In addition, nuclear hormone receptors might bind as monomers to a single hexamer preceded by an A- and T-rich sequence, as shown for Rev-ErbA, retinoic-acid-receptor-related orphan receptor-*α* (ROR*α*), and for nerve-growth-factor-induced clone B (NGFI-B) orphan receptors [100, 125, 126].

### 2.3. Regulation of Transcription

**Figure 2:**
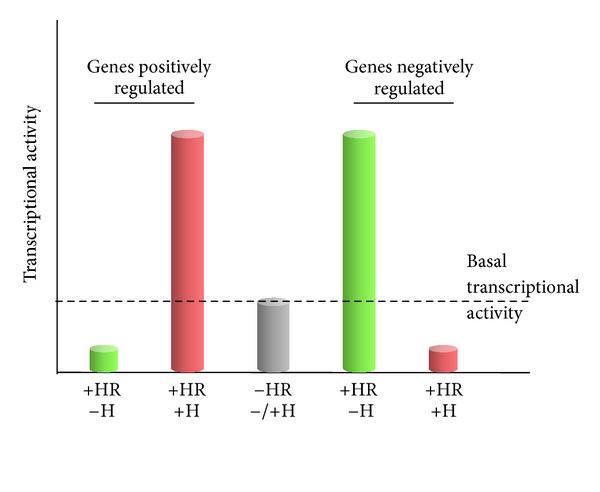
Diagram of transcription regulation by small-molecule hormones. H: hormone, HR: nuclear hormone receptor.

On the basis of the molecular mechanism of action and of the subcellular localization in the absence of ligand, nuclear hormone receptors can be divided into two types. In general, type I receptors preferentially reside in the cytoplasm (in unliganded form) and, while in the nucleus, are most active as homodimers. The best known receptors of this type are family III steroid hormone receptors. Type II receptors, after being synthesized and modified in the cytoplasm, in the presence or absence of their ligand, preferentially translocate to the nucleus, where they are most active as heterodimers. The best known receptors of this type belong to families I and II. Binding of nuclear hormone receptors to DNA might result in transcription activation or in transcription inhibition, and such phenomena result from variable molecular mechanisms. Each hormone has a group of target genes which it activates (positively regulated genes) and a group of genes which it inhibits (negatively activated genes) (Figure 2).

#### 2.3.1. Type I Receptors

**Figure 3:**
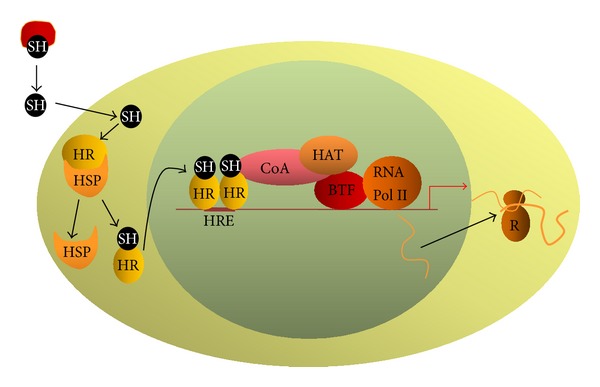
Diagram of type I receptor genomic mode of action. In the circulation, the majority of the hormones form complexes with transporting proteins. The hormone enters the cell by diffusion or is actively transported by specific cell membrane proteins. In the cytoplasm, hormone-free receptors form an inactive complex with heat shock chaperone proteins. Upon hormone binding, the receptor changes its conformation, dissociates from the complex, and translocates into the nucleus. Hormone-activated receptors bind to HREs as homodimers. Recruitment of a coactivator complex possessing a histone acetyltransferase activity results in local chromatin decondensation and increases the accessibility of the promoter for transcription factor. As a result, transcription increases. SH: steroid hormone, HR: nuclear hormone receptor, HSP: heat shock protein, HRE: hormone response element, CoA: coactivator complex, HAT: histone acetyltransferase, BTF: basal transcription factors, RNA Pol II: type II RNA polymerase, and R: ribosome.

In the circulation, steroid hormones are bound to transporting proteins. They enter the cell by diffusion or are actively transported by a cell-membrane-bound transporting proteins. The majority of their nuclear receptors, a classic examples of type I receptors, reside in the cytoplasm forming inactive complexes with various proteins, including heat shock proteins HSP70 and HSP90. Formation of such complexes promotes proper folding of the receptor into a conformation allowing steroid binding [127–131]. Upon hormone binding, receptor conformation changes, and this results in the breakup of the complex. The “activated” receptor translocates to the nucleus thanks to its association with chaperones and importins [132, 133], where it binds to its SREs in the promoters of target genes (Figure 3). It is suggested that intranuclear mobility of steroid receptors, some of the most mobile proteins within the nucleus, depends on the presence of chaperone proteins such as HSP90 [134].

Steroid hormone receptors usually bind to DNA as homodimers. Their preferential SREs are palindromes separated by three neutral nucleotides. Occasionally, they might bind to DNA as monomers; in such a case SRE might consist of only one hexamer and is usually preceded by an A- and T-rich sequence. Binding of the steroid hormone receptor to SRE initiates recruitment of a multiprotein coactivator complex which, by modification of chromatin structure (e.g., histone acetylation by histone acetyltransferases, HATs) and by interaction with the basal transcriptional machinery, activates transcription [135–140] (Figure 3). In addition, steroid hormones acting by their nuclear receptors can potentiate the transactivatory function of other transcription factors [141–143]. 

Inhibition of transcription by steroid hormones and their receptors is a result of a variety of mechanisms, such as hormone-receptor-complex-dependent inhibition of the activity of other transactivators, for example, activator protein 1 (AP1) and NF-*κ*B [144–146]. In this mechanism, binding of the receptor to DNA is not necessary. A number of nSREs are also known. Binding of a hormone-activated or a hormone-free steroid receptor to nSRE leads to the inhibition of transcription mediated either by corepressors bound to hormone-activated receptor or by another group of corepressors bound to hormone-free receptor. Such interaction results in deacetylation of histones exerted by histone deacetylases (HDACs) and in modification of chromatin structure. In turn, chromatin becomes condensed and inaccessible to transcriptional activators [147–151]. Other molecular mechanisms involved in the inhibition of gene transcription *via* nSRE are also known, such as competition for a binding site with transcriptional activators [107, 152–154].

#### 2.3.2. Type II Receptors

Families I and II receptor proteins, synthesized and modified in the cytoplasm, have their NLS exposed so they can translocate to the nucleus in the absence of the hormone. Therefore, both hormone-free and hormone-bound forms of the receptor could be present in the nucleus. Since the conformation of the DNA-binding D domain is stable (independent of the hormone), both receptor forms might bind to the promoter of the target gene; this is why type II receptors are able either to activate or to inhibit transcription of the same gene in a hormone-dependent manner. 

In contrast to type I receptors, type II receptors usually bind to their HREs as heterodimers. Their universal heterodimerization partner is RXR. Heterodimerization with RXR modulates nuclear trafficking of other receptors [155, 156] and increases both affinity of the other receptor to its HRE as well as its transactivation activity [157–160]. Type II receptors can also bind to DNA as heterodimers with nuclear receptors other than RXR, as homodimers and as monomers [111, 161–163]. In such a case, their affinity for DNA might be lower than that of heterodimers with RXR. 

It should be remembered that type II receptors preferentially recognize HREs consisting of two hexamers creating DR, PAL, and IP. In VDR, TR, and RAR heterodimers with RXR, which bind to DR3, DR4, and DR5, respectively, RXR preferentially binds to the first hexamer [164, 165]. On the other hand, in RAR/RXR and PPAR/RXR heterodimers bound to DR1, RXR occupies the second hexamer [166, 167]. The presence of RXR in receptor heterodimers raises the question as to how 9-*cis*-retinoic acid modifies transcription of other hormones' target genes. Most probably it has no influence on the level of activation of triiodothyronine (T3) target genes bound by TR/RXR and of 1*α*,25(OH)_2_D3 target genes bound by VDR/RXR [168, 169]; however, there are reports claiming otherwise [170]. In all-*trans*-retinoic acid target genes bound by the RXR/RAR heterodimer, 9-*cis*-retinoic acid alone does not regulate the activity of such genes, but when both receptors are simultaneously bound to their ligands (9-*cis*-retinoic acid and all-*trans* retinoic acid, respectively), genes are activated synergistically [171]. Finally, when RXR forms heterodimers with a “permissive” partner, such as PPAR, liver X receptor (LXR), or nerve-growth-factor-induced B (NGFI-B) orphan receptor, 9-*cis* retinoic acid can regulate transcription on its own or act synergistically with the ligand of its partner [172, 173].

In addition to HREs mentioned above, type II receptors bind to numerous untypical HREs and to very common nHREs. Binding of the hormone-receptor complex to nHRE results in transcription inhibition, so that the recruitment of corepressors preferentially binding to hormone-activated receptor plays here a major role. 

**Figure 4:**
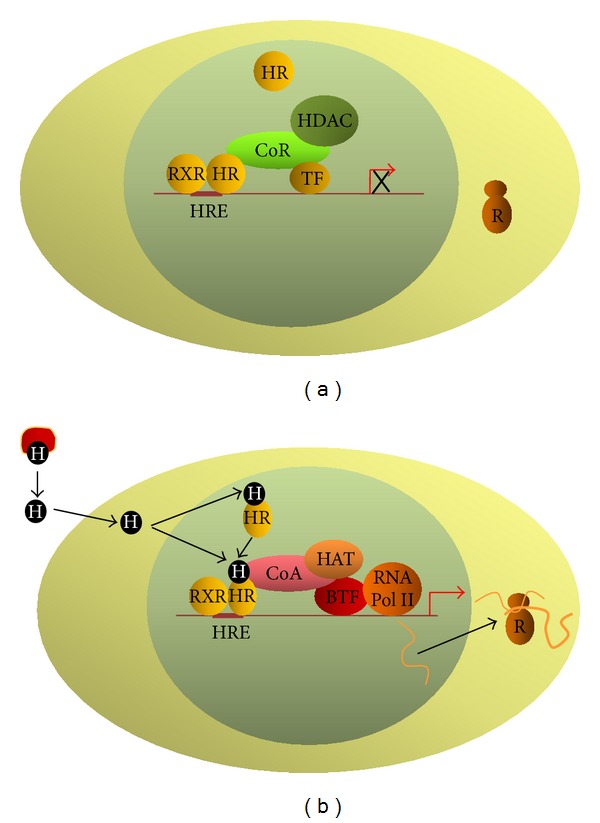
Diagram of type II receptor genomic mode of action. (a) In the absence of the hormone, the receptor binds to HRE as heterodimer with 9-*cis*-retinoic acid receptor. The hormone-free receptor recruits a corepressor complex possessing a histone deacetylase activity. Deacetylation of histones results in chromatin condensation and in transcription inhibition. (b) In the circulation, the hormone forms complexes with transporting proteins. The hormone enters the cell by diffusion or is actively transported by specific cell membrane proteins. The majority of type II receptors reside in the nucleus. Upon hormone binding, the receptor changes its conformation, which results in the dissociation of the corepressor complex and in the binding of the coactivator complex. Histone acetylation by HDAC results in chromatin decondensation, which promotes transcription factor binding to DNA and transcription activation. H: hormone, RH: nuclear hormone receptor, RXR: 9-*cis-*retinoic acid receptor, HRE: hormone response element, CoA: coactivator complex, HAT: histone actyltransferase, CoR: corepressor complex, HDAC: histone deacetylase, TF: transcription factor, BTF: basal transcription factors, RNA Pol II: type II RNA polymerase, and R: ribosome.

**Figure 5:**
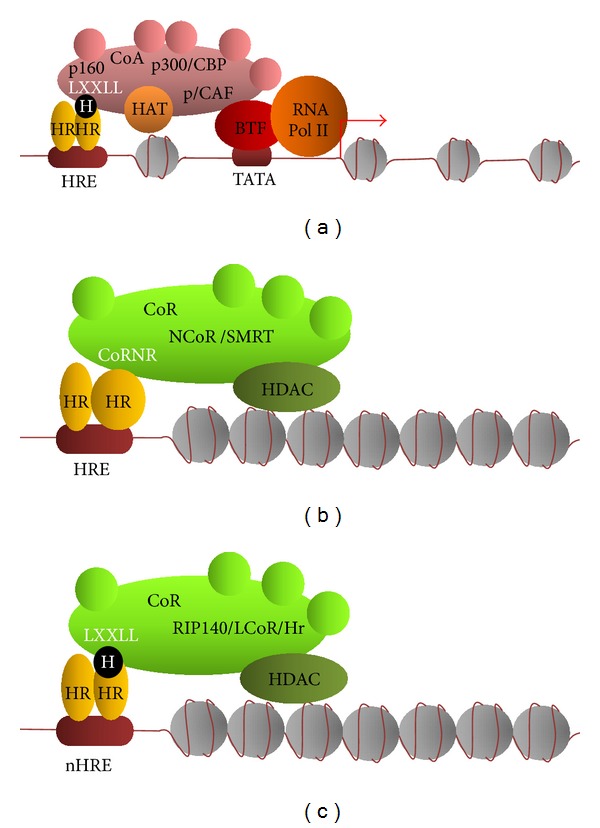
Coactivators, corepressors, chromatin, and regulation of transcription. (a) The coactivator complex consists of many proteins, including proteins with the LXXL (L: leucine, X: any amino acid) motif by which they bind to the hormone-activated nuclear receptor. Histone acetyltransferase activity might be presented by more than one protein of this complex. Decondensation of the chromatin structure upon histone acetylation and a direct interaction of the coactivator complex with basal transcription factors result in transcription activation. (b) In the absence of the hormone, receptor conformation promotes the binding of a multiprotein corepressor complex. Binding of the receptor to this complex occurs by the LXXI/HIXXXI/L (L: leucine, X: any amino acid, I: isoleucine, and H: histidine) motif present in a corepressor protein. The complex includes class I or class II histone deacetylase. Histone deacetylation leads to the condensation of chromatin and, as a result, limits the access of transcription factors to the DNA. As a result, transcription is inhibited. (c) Some corepressor proteins contain the LXXL motif and preferentially bind to the receptors activated by the hormone. Next, by the recruitment of the corepressor complex, including histone deacetylase, they stabilize tight chromatin structure and repress transcription. H: hormone, HR: nuclear hormone receptor, HRE: hormone response element, nHRE: negative hormone response element, CoA: corepressor complex, p160, p300/CBP, and p/CAF: coactivator proteins, LXXL: coactivator protein motif involved in receptor binding, HAT: histone acetyltransferase, CoR: corepressor complex, NCoR/SMRT: classic corepressor complexes, CoRNR: LXXI/HIXXXI/L corepressor protein motif involved in receptor binding, HDAC: histone deacetylase, RIP140/LCoR/Hr: nonclassical corepressor proteins preferentially binding to hormone-activated receptors, BTF: basal transcription factors, and RNA Pol II: type II RNA polymerase.

Hormone target genes with unoccupied HREs are active on the basal level, which depends on the presence of transcription factors other than hormone receptors. In genes positively regulated by the hormone, the binding of a hormone-free receptor heterodimer to HRE leads to the recruitment of a corepressor complex, which, by deacetylation of histones, leads to condensation of chromatin. This, in turn, hampers the binding of transactivators and of basal transcription factors to DNA; as a result, transcription is inhibited below the basal level (Figure 4(a)) [147, 148, 174–176]. However, upon hormone binding to the receptor, conformation of its ligand-binding domain changes; this results in the dissociation of corepressors, in the recruitment of a coactivator complex containing HATs and in transcription activation markedly above the basal level (Figure 5(b)) [177–189]. In genes negatively regulated by the hormone, transcription inhibition occurs as a result of numerous mechanisms; some of them are still not completely known. The inhibition could be indirect, depending on the binding of hormone receptors to a strong transactivator (such as AP1, NF-*κ*B, and p53); such binding results either in a blockage of transactivator's activity or in its binding to DNA [190–192]. In this mechanism, the binding of the receptor to the DNA is not a prerequisite for the inhibition of transcription. In the direct mechanisms, HRE might be present close to or might overlap the binding site for a strong transactivator. Under such circumstances, transcription inhibition is the result either of competition for a binding site, or of binding of the receptor to the transactivator resulting in the repression of its activity [193, 194]. In another direct mechanism, the binding of hormone-activated receptor to nHRE initiates recruitment of specific corepressors preferentially recognizing hormone-bound receptors [149–151, 195–198]. In addition, hormonal receptors bound to nHREs located close to (commonly behind) the transcription start site might affect the binding of type II RNA polymerase to the basal promoter [199].

#### 2.3.3. Interaction of Nuclear Hormone Receptors with Other Proteins

As mentioned above, the biological action of small-molecule hormones depends on their interaction with their receptors, as well as on the interactions of the receptor with DNA and with other proteins. In the genomic mechanism of hormone action, the most important interaction is that of the receptor with coactivators, corepressors, and other transcription factors. On the other hand, in the nongenomic mechanisms, the most crucial role is played by the binding either of the cytoplasmic fraction of nuclear receptors or of hormone itself to extranuclear proteins.

*Interaction of Nuclear Hormone Receptors with Basal Transcription Factors*. Transcription may occur only in the presence of basal transcriptional machinery, a complex consisting of tens of proteins bound to DNA close to the transcription start site. A typical basal promoter contains a TATA box (TATAA/TAA/T) located 20–30 base pairs above TSS, a sequence recognized by TATA-binding protein (TBP). Some promoters do not have this sequence; however, the basal transcriptional machinery binds to such promoters anyway and at a similar distance form TSS as in the case of typical promoters. Binding of TBP to the basal promoter initiates a cascade of binding of other basal transcription factors. TBP together with TBP-binding proteins (TAFs) forms transcription factor IID (TFIID). The next step of preinitiation complex formation is the binding of IIB (TFIIB), IIF (TFIIF), and IIH (TFIIH) transcription factors. Finally, type II RNA polymerase is bound, and transcription is initiated. Nuclear hormone receptors interact with the basal transcription factors not only *via *other proteins (coactivators and corepressors) but also interact with them directly. It has been shown that TR, RXR, RAR, ER, GR, and androgen receptor (AR) might directly bind to TBP, AR and ER—to TFIIF, ER, TR, and VDR—to TFIIB, and so forth [200–205]. It is suggested that such binding might bidirectionally affect (activate or inhibit) the recruitment of the basal transcription factors to the preinitiation complex.

*Interaction of Nuclear Hormone Receptors with Coactivators.* Transfer of information regarding binding of the receptor to HRE and the receptor status (hormone-free or hormone-bound) to the basal transcriptional machinery is usually executed by other proteins that do not bind to DNA but form a functional “bridge”. Such proteins possess various activities. The same coactivator or corepressor complex might bind to several nuclear receptors; some of these complexes might also coregulate transcription initiated by transcription factors of other type. 

The first coactivator cloned in humans was steroid receptor coactivator-1 (SRC-1) [179]. Together with TIF-2 (SRC-2) and TRAM-1 (SRC-3, ACTR, and RAC3), it forms the p160 coactivator family. The p160 proteins are indeed coactivators of many nuclear receptors including GR, ER, PR, VDR, TR, RXR, and PPAR [179, 180, 183, 189]. They contain an LXXLL (L: leucine, X: any amino acid) motif, by which they bind to the ligand-binding domain of the receptor activated by the hormone. Importantly, a specific structure of the receptor, first of all of its AF2 domain, is a prerequisite for such interaction [206]. 

CREB-binding protein (CBP) and p300 possess a histone acetyltransferase activity [207, 208] and are coactivators of various transcription factors, including nuclear hormone receptors [177, 178]. The binding of p300/CBP to the nuclear receptor is hormone dependent and AF2 domain dependent. p300/CBP bind to p160 proteins, to TBP, and to TFIIB basal transcription factors, and, as such, are intermediates between receptors and basal transcriptional machinery. Another coactivator, p300/CBP-associated factor (p/CAF), interacts with p160, p300/CBP, and hormonal nuclear receptors. It also has a histone acetyltransferase activity [182].

Multiprotein complexes containing thyroid-hormone-receptor-associated proteins (TRAP) or vitamin-D-receptor-interacting proteins (DRIP) have been identified [181, 185]. Both complexes are very similar, if not identical, and consists of fourteen-sixteen 70–230 kDa proteins. Their DRIP205/TRAP220/TRIP2 subunit, by the LXXLL motif, interacts with TR, VDR, and other nuclear receptors such as RXR and RAR [209] in a hormone-dependent and a receptor-AF2-domain-dependent manner. Other components of these complexes interact with the basal transcriptional machinery. 

A number of other coactivators interacting with nuclear receptors are known, such as PPAR*γ* coactivator 1 (PGC-1), which also interact with other receptors, for example, with TR [184, 188] and with activating signal cointegrators-1 and -2 (ASC-1 and ASC-2) interacting with SRC-1, p300/CBP, basal transcription factors, and nuclear receptors [186, 187]. 

The formation of a coactivator complex is initiated by the binding of hormone-bound receptor to its HRE. This is followed by the recruitment of the coactivator proteins, which directly bind nuclear receptors and by the binding of other proteins. The final multicomponent complex, by modification of chromatin structure and by interaction with the basal transcriptional machinery, activates transcription of target genes.

*Interaction of Nuclear Hormone Receptors with Corepressors*. Inhibition of transcription is usually achieved by the interaction of the receptor with corepressors [176]. The best known corepressors are nuclear corepressor (NCoR, RIP-13), a large, 270 kDa protein, as well as silencing mediator for retinoic acid and thyroid hormone receptors (SMRT) [147, 148]. Both proteins have several isoforms. Other proteins, such as the small ubiquitous nuclear corepressor (SUN-CoR) and the Alien protein, might also serve as nuclear hormone receptor corepressors [174, 175]. The motif that allows NCoR and SMRT to bind to the receptor is LXXI/HIXXXI/L (L: leucine, X: any amino acid, I: isoleucine, and H: histidine) [210, 211]. NCoR and SMRT bind to the families I and II nuclear receptors, to ER and to PR (but not to other members of family III) bound to a specific antagonists, and to some orphan receptors. They also bind to other proteins, including HDACs [212, 213].

Recent developments identified a heterogeneous group of corepressors of a new type. What makes them unique among corepressors is the fact that they bind to the receptor activated by the hormone. The group includes receptor-interacting protein 140 (RIP140) and ligand-dependent corepressor (LCoR). They bind to a various ligand-bound receptors, including ER, GR, PR, and VDR, *via *the coactivator-specific LXXLL motif, but recruit HDAC proteins and other corepressors [149–151]. 

Hairless protein (Hr) contains both the hormone-activated-receptor-binding LXXLL motif and a CoRNR box—a sequence mediating the binding of the corepressor to the hormone receptor. When it interacts with ligand-bound ROR, it utilizes the LXXLL motif, whereas when it interacts with VDR, it likely utilizes another domain. On the other hand, Hr interacts with hormone-free TR as a typical corepressor, utilizing the CoRNR box and recruiting HDAC [195–197]. 

The preferentially expressed antigen in melanoma (PRAME) is expressed in various cancers, but in healthy tissues it is present only in testes, ovaries, endometrium, and adrenal glands. PRAME contains the LXXLL motif and selectively inhibits transcription in the presence of all all-*trans*-retinoic-acid-bound RAR isoforms. It likely executes this inhibition by recruiting other corepressors [198]. 

The repressor of estrogen activity (REA) binds to the ER-agonist (e.g., 17*β*-estradiol) and to the ER-antagonist (e.g., tamoxifen) complexes. By doing so in the presence of agonist, it inhibits the activity of target gene, while in the presence of antagonist it magnifies its action [214]. The suppression by REA is a result of the competition with coactivators for binding to ER, as well as of the recruitment of HDAC and of chromatin modification. 

Metastasis-associated factor 1 (MTA1) is another corepressor preferentially binding to a ligand-activated ER [215]. It inhibits the expression of estrogen target genes by competing with coactivators for the binding to the receptor, by recruiting HDAC, and by chromatin modification.

A group of corepressors that might bind both liganded and nonliganded hormone receptors is also known. For example, the NR-binding SET domain containing protein 1 (NSD1) possesses separate domains: one that binds hormone-free receptors TR and RAR (NID^−L^) and another that binds hormone-bound TR, RXR, ER, and RAR receptors (NID^+L^) [216].

*Interaction of Hormonal Nuclear Receptors with Other Transcription Factors.* Natural HREs are located relatively close to TSS or in more distant regulatory elements, and binding sites for other transcription factors are usually located nearby. Such proximity permits interaction between nuclear receptors and these transcription factors, leading either to the suppression of gene activity (as described above) or to its additive or synergistic activation. The binding of nuclear receptors to other transcription factors might also occur in a DNA-binding-independent manner. In fact, the binding of all known nuclear hormone receptors to the transcription factors has been reported; the best known examples are the binding of TR to p53, GR and PR to Oct-1, GR to AP-1 and to NF-*κ*B, PPAR to NF-*κ*B, AP-1, and to STAT [217–221]. 

#### 2.3.4. Nuclear Hormone Receptors and Chromatin

A nucleosome consists of eight histone molecules (two of each H2A, H2B, H3, and H4). Their N-terminal ends (tails) protrude from the compact nucleosome body. Epigenetic modifications of amino acids forming such tails play a marked role in chromatin organization. Increased acetylation relieves compact chromatin, which results in an exposure of the transcription-factor-binding sites and their increased accessibility leading to transcription activation. On the other hand, deacetylation of histone tails leads to the formation of a compact chromatin. As a result, transcription-factor-binding sites become inaccessible to transactivators, and the gene becomes transcriptionally inactive. Such a mechanism of modification of chromatin structure is utilized by nuclear hormone receptors, which, as mentioned above, interact with coactivators and corepressors. p160 and p300/CBP coactivators themselves possess HAT activity and form complexes with other HAT proteins, such as p/CAF. On the other hand, corepressor proteins recruit class I and class II HDAC proteins to the corepressor complex. The binding of a ligand-activated receptor to HRE initiates the formation of a coactivator complex, which, thanks to the HAT activity, increases histone acetylation and induces local decondensation of chromatin (Figure 5(a)). On the other hand, the binding of a hormone-free receptor to HRE initiates the formation of a corepressor complex, which, thanks to its HDAC activity, induces local condensation of chromatin (Figure 5(b)). Finally, the corepressor complex and its HDAC activity are utilized by the specific corepressor proteins described above, which bind to a hormone-activated receptor and inhibit transcription of the target gene (Figure 5(c)).

## 3. The Nongenomic Mechanisms of Action of Small-Molecule Hormones 

**Figure 6:**
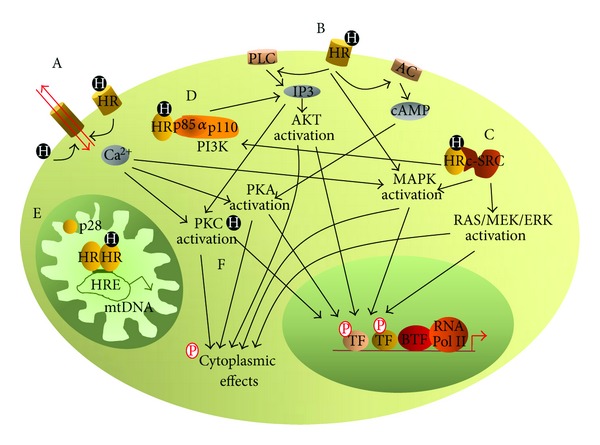
Simplified diagram of nongenomic mechanisms of action of small-molecule hormones. All nongenomic mechanisms activate numerous transduction pathways, and, by a series of phosphorylation events of cytoplasmic and of nuclear proteins, modify cell function. (A) Interaction of the hormone with either cell membrane receptor or directly with membrane phospholipids modifies the function of ion channels. (B) Activation of phospholipase (C) initiated by the hormone-activated cell membrane receptor, and of adenylate cyclase (and, most possibly, of other enzymes) stimulates production of secondary messengers. (C) Activation of c-SRC by the hormone-activated nuclear receptor. (D) The binding of the hormone-activated nuclear receptor to the phosphatidylinositol 3 kinase p85*α* subunit activates this enzyme and results in an increased synthesis of inositol triphosphate. (E) In mitochondria, small-molecule hormones acting *via* their nuclear receptors or by their shorter (mitochondrial) isoforms regulate transcription of mitochondrial DNA. In addition, interaction of some hormones alone or of hormone-receptor complexes with mitochondrial proteins stimulates thermogenesis. (F) The binding of the hormone activates protein kinase. (C) HR: various types of hormone receptors, Ca^2+^: calcium ion, p85*α*, p110: phosphatidylinositol 3 kinase subunits, PI3K: phosphatidylinositol 3 kinase, IP3: inositol triphosphate, PLC: phospholipase. (C) AC: adenylate cyclase, cAMP: cyclic AMP, AKT: protein kinase. (B) c-SRC: tyrosine kinase, PKA: protein kinase. (A) PKC: protein kinase. (C) MAPK: mitogen-activated protein kinase, RAS/MEK/ERK: protein kinases, p28: shortest isoform of TR*α*, mtDNA: mitochondrial DNA, HRE: hormone response element, TF: transcription factor, BTF: basal transcription factors, and RNA Pol II: type II RNA polymerase.

Fast biological effects of hormones, just seconds or minutes after hormone administration, have already been described several dozen years ago. The rapidity of biological response and its independence from transcription and from translation suggested that the genomic mechanism of hormone action is not involved; therefore, this mechanism was called nongenomic or extragenomic. The nongenomic mechanisms of hormone action are multiple, variable, and only partially known (Figure 6).

### 3.1. Nongenomic Mechanisms of Hormone Action Induced by the Interaction of Hormones with Membrane and Cytoplasmic Receptors

Steroid and nonsteroid small-molecule hormones bind to various proteins localized outside the nucleus and activate transduction pathways leading to a fast biological response. The presence of binding sites in the cell membrane was proved for all major representatives of these hormones; however, in many cases the identity of the binding protein remains unknown. In addition, it is likely that such hormones have more than one type of membrane receptors. In the case of receptors already identified, their mode of action is by and large only partially resolved. 

Just next to the cell membrane or directly in it, usually within caveolae (a bubble-like, 50–100 nm invaginations of the cell membrane), proteins identical to the nuclear receptors for glucocorticoids, estrogen, androgen, and vitamin D, have been identified [222–225]. It is then plausible that nuclear receptors of other small-molecule hormones are present close to or in the cell membrane. Some small-molecule hormones bind to other than nuclear receptor-like cell membrane proteins. For example, the integrin receptor *α*V*β*3 plays a role of cell membrane receptor for thyroxin (T4) [226]. mPR*α*, mPR*β*, mPR*γ*, mPR*δ*, and mPR*ε* cell membrane receptors for progesterone possess seven transmembrane domains (some authors even suggest the presence of eight such domains) and interact with G proteins [227, 228]. The G-protein-interacting cell membrane receptor for steroid-hormone-binding protein (SHBG) binds androgens with higher and estrogens with lower affinity. The prerequisite for signal transduction from the hormone to the cell interior by this receptor is the binding of a hormone-free SHBG first, followed by hormone binding [229, 230]. *γ*-Aminobutyric acid A (GABA_A_) receptor serves as the cell membrane receptor for neurosteroids [231].

#### 3.1.1. Nuclear Hormone Receptor Targeting at the Membrane

The best studied is membrane targeting of ER. It is induced by palmitoylation of cysteine 447 [232], a modification increasing protein hydrophobicity and, therefore, facilitating protein association with lipid bilayer. Truncated 46 kDa variant of ER*α* is preferentially palmitoylated and enriched in the cell membranes [233]; it is suggested that it might be more active than full-length receptor [234]. Another membrane-localized variant of ER*α*, ER*α*-36, is also functionally active [235]. Palmitoylation of ER is promoted by HSP27 [236]. 

Enzymes identified as palmitoylacyltransferases for sex hormone receptors are DHHC-7 and DHHC-21 proteins [237]. A highly conserved 9-amino acid motif (FVCLKSIIL in ER*α*) that is crucial for palmitoylation and membrane localization has been identified in the ligand-binding domains of ER*α*, ER*β*, PR*α*, PR*β*, GR, and AR [238]. TR*α* and TR*β* possess a motif (LPCEDQIIL) that slightly differs from the one described above, but presumably, it is also involved in the receptor palmitoylation and membrane targeting. Notably, MR, PPARs, and RAR do not have any sequence resembling this motif [238]. 

Translocation of nuclear hormone receptors to the membrane is also induced in the presence of the respective ligand; this was shown for ER*α*- and 17*β*-estradiol [239] and for VDR and 1*α*,25(OH)_2_D3 [240]. 

In the cell membrane, nuclear hormone receptors interact with caveolae-specific proteins; for example, ER*α* and AR physically interact with Caveolin-1 [234, 241], while VDR binds to Caveolin-3 [242]. Binding to caveolins is required for membrane localization of the receptor [234]. Furthermore, binding to caveolins allows hormone receptors to initiate fast, specific nongenomic response to hormonal stimulus.

#### 3.1.2. Induction of Transduction Pathways

Upon binding to the cell membrane receptors, small-molecule hormones activate various transduction pathways by a receptor-type-dependent mechanism. By activation of phospholipase C (PLC) and generation of the secondary messenger inositol 1,4,5-trisphosphate (IP3), they might activate the cell membrane and the sarcoplasmic reticulum (the most important Ca^2+^ storage) ion channels. Such activation leads to the increase of intracellular concentration of Ca^2+^, another secondary messenger crucial for many cellular functions. Ca^2+^ activates, among others, RAS/RAF/MEK/ERK kinases, protein kinase C (PKC), and protein kinase A (PKA). As a result, activated kinases phosphorylate and activate numerous cytoplasmic and nuclear proteins, including hormonal receptors, transcription factors and coactivators. This, in turn, modulates various biological processes in the cytoplasm and influences transcription of genes regulated by newly phosphorylated hormone receptors and transcription factors. Cell-membrane-located small-molecule hormone receptors interacting with G proteins might also activate adenylate cyclase, which results in the generation of yet another secondary messenger, cAMP, and in the activation of cAMP-dependent proteins, such as PKA, and of their substrates [243–247]. By nongenomic mechanisms, small-molecule hormones also regulate the activity of ion channels, influencing cross-membrane movement of Na^+^, H^+^, Cl^−^, and of K^+^ [245, 248, 249]. 

Small-molecule hormones also bind to the proteins present in the cytoplasm; commonly, such proteins are cytoplasmic fractions of nuclear receptors. Upon hormone binding, the receptor interacts with numerous proteins, elements of various signal transduction pathways which, as described above, might be also activated by hormones on a “higher” level, namely, that of a cell membrane receptor. For example, hormone-activated TR, ER, and RAR bind to a p85*α* subunit of phosphatidylinositol 3 kinase. Activated kinase increases production of IP3 which, in turn, activates the mitogen-activated protein kinase (MAPK) pathway [250–252]. Hormone-activated AR, PR, and ER bind to SH3 or to SH2 subunit of c-SRC tyrosine kinase localized close to the cell membrane. Such binding activates c-SRC which, subsequently, activates MAPK and RAS/RAF/MEK/ERK pathways leading to the phosphorylation of various cytoplasmic and nuclear receptors [253–255].

### 3.2. Nuclear Hormone Receptor Binding to Calmodulin

Of note is nuclear hormone receptors' binding to CaM, being an example of cross-talking of hormonal signaling with other signal transduction pathways. Such binding has been proved for ER*α* (but not for ER*β*), AR, and orphan receptor ERR*γ*, among others [85–87, 256–258]. It results in the increased stability of the receptor due to CaM-dependent protection from degradation [85–87]. CaM facilitates dimerization of ER*α* in the absence of 17*β*-estradiol [86]. The binding profoundly affects receptor function: CaM is required for normal transactivation by ER*α* since its elimination or blockage by antagonists prevents 17*β*-estradiol from inducing transcriptional activity of this receptor [256, 257]. Similarly, CaM stimulates transcriptional activity of AR (since its antagonist W-7 blocks AR-dependent expression of prostate-specific antigen) and of ERR*γ* [258, 259]. 

### 3.3. Hormone Binding to Nonreceptor Proteins

Small-molecule hormones could also bind to another, nonreceptor type cytoplasmic proteins. For example, 1*α*,25(OH)_2_D3, dehydroepiandrosterone (DHEA), and dexamethasone bind to PKC*α*, PKC*γ*, and PKC*ε* isoforms of PKC, which results in enzyme activation. In addition, PKC*α* isoform is also directly activated by aldosterone and by 17*β*-estradiol, while PKC*δ* isoform is activated by 17*β*-estradiol [260, 261].

### 3.4. Small-Molecule Hormones Action in Mitochondria

Small-molecule hormones modulate the function of mitochondria by a number of mechanisms. One of them is based on the action of their nuclear receptors as transcription factors. Each mitochondrion has multiple copies of its own DNA (mtDNA) encoding 37 genes, including genes for 13 proteins involved in oxidative phosphorylation. A shortened isoform of TR (mtTR*α*1, so-called p43), RXR*α* (mtRXR*α*), and PPAR*γ*2 (mtPPAR*γ*2), as well as full-length GR, ER*α*, and ER*β* receptors are present in mitochondria [109, 262–265], where they form dimers such as mtRXR*α*/p43, mtPPAR*γ*2/p43, GR/GR, and ER/ER or couple with other transcription factors. It has been shown that glucocorticoids, T3 and 17*β*-estradiol, acting by their mitochondrial receptors bound to mitochondrial HREs, activate the transcription of mtDNA, leading to an increased activity of oxidative phosphorylation.

Another mechanism of small-molecule hormones action in mitochondria is based on their interactions with other proteins. For example, diiodothyronine (T2) binds to the Va subunit of cytochrome *c* oxidase and activates this enzyme [266]. Adenine nucleotide translocase (ANT) binds all-*trans*-retinoic acid [267]. 

In addition, the shortest isoform of TR*α*1, p28, is bound to the internal mitochondrial membrane [263], where, most likely, it stimulates the function of ANT and of uncoupling proteins (UCPs) [263, 268]. Orphan nuclear receptor Nur77 mediates apoptosis by interaction with Bcl-2 and by induction of cytochrome *c* release [269]. 

Small-molecule hormones acting in mitochondria regulate Ca^2+^ wave activity in this organelle, as shown in the case of estrogens and T3 [270, 271]. 

Finally, hormonal receptors can directly bind to mitochondrial membranes and modify membrane potential, as shown, for example, for stress-activated GR [272].

### 3.5. Interaction of Hormonal Nuclear Receptors with RNA

It has been shown that RAR*α* molecules present in the cytoplasm can bind mRNA *via* the C-terminal F domain, which recognizes a specific sequences in the target mRNA. Such a mechanism was described for mRNA encoding neuronal GluR1 protein, a subunit of the glutaminergic receptor. The binding of GluR1 mRNA by a hormone-free receptor results in the inhibition of translation. The binding of all-*trans*-retinoic acid induces the change of receptor conformation and decreases its affinity for mRNA; as a result the receptor dissociates from mRNA [273].

### 3.6. Direct Interaction of Small-Molecule Hormones with Membranes

A very rapid effects of androgens, progesterone, glucocorticoids, and other steroid hormones, evident just a few seconds after hormone administration, might be a result of a nonspecific, nongenomic mechanism of small-molecule hormones action, based on their interactions with lipid bilayers. Lipophilic steroid hormone molecules could directly bind to membrane phospholipids and, by doing so, modulate their function. This, in turn, influences the function of membrane proteins such as the calcium pump and other channel proteins, leading to an immediate transport modification of various ions. Nonspecific binding of steroid hormones to a mitochondrial membrane might increase proton leak [274, 275]. 

## 4. Human Pathologies Associated with Receptor Abnormalities

Medical conditions associated with out-of-range level of small-molecule hormones are known for decades, relatively common, and have been exhaustively described in numerous handbooks and articles. In contrast, much less is known about diseases initiated by abnormalities of the receptor. They are uncommon, with a wide range of signs and symptoms of variable severity (related to both the type and site of genetic error within the receptor-encoding gene or related genes) that might mimic signs and symptoms of other diseases (e.g., resistance to thyroid hormone might be erroneously diagnosed as hyperthyroidism). Detailed description of these diseases exceeds the scope of this paper; however, in Table 2 the reader can find a comprehensive summary and references to the review and original articles regarding selected human hormone-receptor-related pathologies.

Hormone-receptor-related diseases constitute an important diagnostic challenge. Among them, a monogenic diseases arising due to mutation are the easiest to diagnose, provided that a candidate gene is identified and its sequencing shows mutation. It is much more difficult, though, to evaluate the influence of altered expression or function (e.g., due to the abnormal posttranslational modifications) of the receptor on the phenotype, especially of multifactorial diseases such as obesity, insulin resistance, atherosclerosis, cardiovascular disease, cancer, neurodegeneration, and so forth the Diagnostic problems are the reasons why hormone receptor dysfunctions commonly remain undiagnosed and untreated. However, the importance of such dysfunctions in pathophysiology of both rare and common diseases fully justifies the efforts to elucidate the molecular mechanisms of action of these receptors. Importantly, identification of these mechanisms is crucial for designing new targeted therapeutic strategies.

## 5. Conclusion

Small-molecule hormones, usually of quite simple chemical structure, have an enormously wide range of biological functions. The effects of their action are due to their interaction with various receptors, which, by further interaction with other proteins or with DNA, activate various signal transduction pathways or regulate the activity of numerous target genes. Even though our knowledge regarding these nongenomic and genomic mechanisms is already impressive, a lot of information regarding, first of all, their interdependence still awaits elucidation.

## Figures and Tables

**Table 1:** Selected representatives of the nuclear receptor superfamily.

Family	Receptor	Ligand
I	Triiodothyronine receptor (TR)	Triiodothyronine
	Retinoic acid receptor (RAR)	All*-trans*-retinoic acid
	Vitamin D receptor (VDR)	1*α*,25(OH)_2_D3
	Peroxisome-proliferator-activated receptor (PPAR)	Polyunsaturated fatty acids, benzopyran, eicosanoids, 15-deoxy-12,41-prostaglandin J_2_, thiazolidinediones, other
	Reverse-ErbA (Rev-ErbA)	Unknown
	Retinoic-acid-receptor-related orphan receptor (ROR)	Unknown
	Liver X receptor (LXR)	Oxysterols

II	9-*cis-*Retinoic acid receptor (RXR)	9-*cis*-Retinoic acid
	Hepatocyte nuclear factor-4 (HNF-4)	Acyl-CoA thioesters

III	Estrogen receptor (ER)	17*β*-estradiol
	Androgen receptor (AR)	Androgens
	Progesterone receptor (PR)	Progesterone
	Glucocorticoid receptor (GR)	Glucocorticoids
	Mineralocorticoid receptor (MR)	Mineralocorticoids, glucocorticoids

IV	Nerve-growth-factor-induced clone-B (NGFI-B)	Unknown

V	Steroidogenic factor-1 (SF-1)	Oxysterols

VI	Germ cell nuclear factor (GCNF)	Unknown

0	Heterodimerization small partner (HSP)	Unknown

**Table 2:** Selected human pathologies associated with hormone receptors.

Receptor	Pathology
TR	Mutation-related generalized and pituitary resistance to thyroid hormone [1–4]; mutations and/or altered expression in various cancers [5–9]
RAR	Translocation in acute promyelocytic leukemia [10]; reduced expression in cancers [11]; altered signaling in neurological and psychiatric diseases [12]
VDR	Mutation-related resistance to 1*α*,25(OH)_2_D3/hereditary vitamin D resistant rickets [13–15]; polymorphisms in osteoporosis [16]; mutations in alopecia [17]; altered expression and polymorphisms in various cancers [18–20]; altered function in inflammation [21]; altered function in liver pathology [22]
PPAR	Mutations in insulin resistance in nonobese [23]; mutations in familial partial lipodystrophy [23–25]; excessive phosphorylation in insulin resistance and obesity [26]; mutations in cancers, low expression in cancers with poor prognosis [27]; alterations in atherosclerosis, inflammation, and osteoarthritis [28–30]
RXR	Polymorphisms in colorectal cancer and in metabolic diseases [31–33]
ER	Mutations and altered expression in breast cancer [34–36]; altered posttranslational modifications in breast cancer [36, 37]; overexpression in endometriosis [38]; polymorphisms in ovulatory dysfunction [39]; impaired function in metabolic diseases [40]
AR	Mutation-related androgen insensitivity syndrome [41–43]; overexpression, mutations, CAG repeat extension, excessive receptor phosphorylation in prostate cancer [26, 41, 44–46]; CAG repeat extension in spinal and bulbar muscular atrophy [45, 47]; mutations and the *AR *gene trinucleotide repeat variations in male infertility [48]; mental disorders [41]
PR	Lack of expression in breast cancer [35, 36]; decreased expression in endometriosis [49]; altered expression in testis of infertile men [50]
GR	Mutation-related glucocorticoid resistance [51, 52]; polymorphisms in tissue-specific sensitivity to glucocorticoids and hypersensitivity to glucocorticoids [52]; polymorphisms in depression [53]
MR	Mutations in mineralocorticoid resistance syndrome (pseudohypoaldosteronism type 1) [54]; polymorphisms in depression [53]; mutation in severe hypertension [55]

## References

[1] Suzuki S, Shigematsu S, Inaba H, Takei M, Takeda T, Komatsu M (2011). Pituitary resistance to thyroid hormones: pathophysiology and therapeutic options. *Endocrine*.

[2] Ferrara AM, Onigata K, Ercan O, Woodhead H, Weiss RE, Refetoff S (2012). Homozygous thyroid hormone receptor b gene mutations in resistance to thyroid hormone: three new cases and review of the literature. *Journal of Clinical Endocrinology and Metabolism*.

[3] Kannan S, Safer JD (2012). Finding the right balance between resistance and sensitivity: a review of the cardiac manifestations of the syndrome of resistance to thyroid hormone and the implications for treatment. *Endocrine Practice*.

[4] van Mullem A, van Heerebeek R, Chrysis D (2012). Clinical phenotype and mutant TRa1. *The New England Journal of Medicine*.

[5] Turowska O, Nauman A, Pietrzak M (2007). Overexpression of E2F1 in clear cell renal cell carcinoma: a potential impact of erroneous regulation by thyroid hormone nuclear receptors. *Thyroid*.

[6] Lu C, Cheng SY (2011). Extranuclear signaling of mutated thyroid hormone receptors in promoting metastatic spread in thyroid carcinogenesis. *Steroids*.

[7] Rosen MD, Privalsky ML (2011). Thyroid hormone receptor mutations in cancer and resistance to thyroid hormone: perspective and prognosis. *Journal of Thyroid Research*.

[8] Lu C, Mishra A, Zhu YJ, Meltzer P, Cheng SY (2011). Global expression profiling reveals gain-of-function oncogenic activity of a mutated thyroid hormone receptor in thyroid carcinogenesis. *American Journal of Cancer Research*.

[9] Rosen MD, Chan IH, Privalsky ML (2011). Mutant thyroid hormone receptors (TRs) isolated from distinct cancer types display distinct target gene specificities: a unique regulatory repertoire associated with two renal clear cell carcinomas. *Molecular Endocrinology*.

[10] Chen Z, Wang ZY, Chen SJ (1997). Acute promyelocytic leukemia: cellular and molecular basis of differentiation and apoptosis. *Pharmacology and Therapeutics*.

[11] Olasz J, Juhász A, Remenár E (2007). RAR*β*2 suppression in head and neck squamous cell carcinoma correlates with site, histology and age. *Oncology Reports*.

[12] van Neerven S, Kampmann E, Mey J (2008). RAR/RXR and PPAR/RXR signaling in neurological and psychiatric diseases. *Progress in Neurobiology*.

[13] Nguyen TM, Adiceam P, Kottler ML (2002). Tryptophan missense mutation in the ligand-binding domain of the vitamin D receptor causes severe resistance to 1,25-dihydroxyvitamin D. *Journal of Bone and Mineral Research*.

[14] Aljubeh JM, Wang J, Al-Remeithi SS, Malloy PJ, Feldman D (2011). Report of two unrelated patients with hereditary vitamin D resistant rickets due to the same novel mutation in the vitamin D receptor. *Journal of Pediatric Endocrinology and Metabolism*.

[15] Malloy PJ, Zhou Y, Wang J, Hiort O, Feldman D (2011). Hereditary vitamin D-resistant rickets (HVDRR) owing to a heterozygous mutation in the vitamin D receptor. *Journal of Bone and Mineral Research*.

[16] Massart F, Marcucci G, Brandt ML (2008). Pharmacogenetics of bone treatments: the VDR and ER*α* gene story. *Pharmacogenomics*.

[17] Malloy PJ, Feldman D (2011). The role of vitamin D receptor mutations in the development of alopecia. *Molecular and Cellular Endocrinology*.

[18] Köstner K, Denzer N, Müller CSL, Klein R, Tilgen W, Reichrath J (2009). The relevance of Vitamin D Receptor (VDR) gene polymorphisms for cancer: a review of the literature. *Anticancer Research*.

[19] Field S, Newton-Bishop JA (2011). Melanoma and vitamin D. *Molecular Oncology*.

[20] Welsh J (2012). Cellular and molecular effects of vitamin D on carcinogenesis. *Archives of Biochemistry and Biophysics*.

[21] Wu S, Sun J (2011). Vitamin D, vitamin D receptor, and macroautophagy in inflammation and infection. *Discovery Medicine*.

[22] Zúñiga S, Firrincieli D, Housset C, Chignard N (2011). Vitamin D and the vitamin D receptor in liver pathophysiology. *Clinics and Research in Hepatology and Gastroenterology*.

[23] Visser ME, Kropman E, Kranendonk ME (2011). Characterisation of non-obese diabetic patients with marked insulin resistance identifies a novel familial partial lipodystrophy-associated PPAR*γ* mutation (Y151C). *Diabetologia*.

[24] Jeninga EH, Kalkhoven E (2010). Central players in inherited lipodystrophies. *Trends in Endocrinology and Metabolism*.

[25] Vigouroux C, Caron-Debarle M, Le Dour C, Magré J, Capeau J (2011). Molecular mechanisms of human lipodystrophies: from adipocyte lipid droplet to oxidative stress and lipotoxicity. *International Journal of Biochemistry and Cell Biology*.

[26] Anbalagan M, Huderson B, Murphy L, Rowan BG (2012). Post-translational modifications of nuclear receptors and human disease. *Nuclear Receptor Signaling*.

[27] Robbins GT, Nie D (2012). PPARg, bioactive lipids, and cancer progression. *Frontiers in Bioscience*.

[28] Wang N, Yin R, Liu Y, Mao G, Xi F (2011). Role of peroxisome proliferator-activated receptor-*γ* in atherosclerosis: an update. *Circulation Journal*.

[29] Martin H (2010). Role of PPAR-gamma in inflammation. Prospects for therapeutic intervention by food components. *Mutation Research*.

[30] Fahmi H, Martel-Pelletier J, Pelletier JP, Kapoor M (2011). Peroxisome proliferator-activated receptor gamma in osteoarthritis. *Modern Rheumatology*.

[31] Jacobs ET, Martínez ME, Campbell PT (2010). Genetic variation in the retinoid X receptor and calcium-sensing receptor and risk of colorectal cancer in the Colon Cancer Family Registry. *Carcinogenesis*.

[32] Hsieh CH, Pei D, Hung YJ, Hsiao FC (2011). Association between retinoid-X receptor g genetic polymorphisms and diabetic retinopathy. *Genetics and Molecular Research*.

[33] Shi H, Yu X, Li Q (2012). Association between PPAR*γ* and RXR*α* gene polymorphism and metabolic syndrome risk: a case-control study of a Chinese Han population. *Archives of Medical Research*.

[34] Barone I, Brusco L, Fuqua SAW (2010). Estrogen receptor mutations and changes in downstream gene expression and signaling. *Clinical Cancer Research*.

[35] Valentin MD, da Silva SD, Privat M, Alaoui-Jamali M, Bignon YJ (2012). Molecular insights on basal-like breast cancer. *Breast Cancer Research and Treatment*.

[36] Eiermann W, Bergh J, Cardoso F (2012). Triple negative breast cancer: proposals for a pragmatic definition and implications for patient management and trial design. *Breast*.

[37] Le Romancer M, Poulard C, Cohen P, Sentis S, Renoir JM, Corbo L (2011). Cracking the estrogen receptor's posttranslational code in breast tumors. *Endocrine Reviews*.

[38] Bulun SE, Monsavais D, Pavone ME (2012). Role of estrogen receptor *β* in endometriosis. *Seminars in Reproductive Medicine*.

[39] Drummond AE, Fuller PJ (2012). Ovarian actions of estrogen receptor b: an update. *Seminars in Reproductive Medicine*.

[40] Faulds MH, Zhao C, Dahlman-Wright K, Gustafsson JÅ (2012). The diversity of sex steroid action: regulation of metabolism by estrogen signaling. *Journal of Endocrinology*.

[41] Rajender S, Singh L, Thangaraj K (2007). Phenotypic heterogeneity of mutations in androgen receptor gene. *Asian Journal of Andrology*.

[42] Galani A, Kitsiou-Tzeli S, Sofokleous C, Kanavakis E, Kalpini-Mavrou A (2008). Androgen insensitivity syndrome: clinical features and molecular defects. *Hormones*.

[43] Hughes IA, Davies JD, Bunch TI, Pasterski V, Mastroyannopoulou K, MacDougall J (2012). Androgen insensitivity syndrome. *The Lancet*.

[44] Saraon P, Jarvi K, Diamandis EP (2011). Molecular alterations during progression of prostate cancer to androgen independence. *Clinical Chemistry*.

[45] Kumar R, Atamna H, Zakharov MN, Bhasin S, Khan SH, Jasuja R (2011). Role of the androgen receptor CAG repeat polymorphism in prostate cancer, and spinal and bulbar muscular atrophy. *Life Sciences*.

[46] Bergerat JP, Céraline J (2009). Pleiotropic functional properties of androgen receptor mutants in prostate cancer. *Human Mutation*.

[47] Katsuno M, Banno H, Suzuki K, Adachi H, Tanaka F, Sobue G (2010). Clinical features and molecular mechanisms of spinal and bulbar muscular atrophy (SBMA). *Advances in Experimental Medicine and Biology*.

[48] Gottlieb B, Lombroso R, Beitel LK, Trifiro MA (2005). Molecular pathology of the androgen receptor in male (in )fertility. *Reproductive BioMedicine Online*.

[49] Bulun SE, Cheng YH, Pavone ME (2010). Estrogen receptor-*β*, estrogen receptor-*α*, and progesterone resistance in endometriosis. *Seminars in Reproductive Medicine*.

[50] Abid S, Gokral J, Maitra A (2008). Altered expression of progesterone receptors in testis of infertile men. *Reproductive BioMedicine Online*.

[51] van Rossum EFC, Lamberts SWJ (2006). Glucocorticoid resistance syndrome: a diagnostic and therapeutic approach. *Best Practice and Research*.

[52] Nicolaides NC, Galata Z, Kino T, Chrousos GP, Charmandari E (2010). The human glucocorticoid receptor: molecular basis of biologic function. *Steroids*.

[53] DeRijk RH, de Kloet ER (2008). Corticosteroid receptor polymorphisms: determinants of vulnerability and resilience. *European Journal of Pharmacology*.

[54] Kino T, Chrousos GP (2004). Glucocorticoid and mineralocorticoid receptors and associated diseases. *Essays in Biochemistry*.

[55] Rafestin-Oblin ME, Souque A, Bocchi B, Pinon G, Fagart J, Vandewalle A (2003). The severe form of hypertension caused by the activating S810L mutation in the mineralocorticoid receptor is cortisone related. *Endocrinology*.

[56] Nuclear Receptors Nomenclature Committee (1999). A unified nomenclature system for the nuclear receptor superfamily. *Cell*.

[57] Escriva H, Bertrand S, Laudet V (2004). The evolution of the nuclear receptor superfamily. *Essays in Biochemistry*.

[58] Germain P, Staels B, Dacquet C, Spedding M, Laudet V (2006). Overview of nomenclature of nuclear receptors. *Pharmacological Reviews*.

[59] Rochette-Egly C, Gaub MP, Lutz Y, Ali S, Scheuer I, Chambon P (1992). Retinoic acid receptor-*β*: immunodetection and phosphorylation on tyrosine residues. *Molecular Endocrinology*.

[60] Juge-Aubry CE, Hammar E, Siegrist-Kaiser C (1999). Regulation of the transcriptional activity of the peroxisome proliferator-activated receptor by phosphorylation of a ligand-independent trans-activating domain. *Journal of Biological Chemistry*.

[61] Nordzell M, Aarnisalo P, Benoit G, Castro DS, Perlmann T (2004). Defining an N-terminal activation domain of the orphan nuclear receptor Nurr1. *Biochemical and Biophysical Research Communications*.

[62] Rajbhandari P, Finn G, Solodin NM (2012). Regulation of estrogen receptor N-terminus conformation and function by peptidyl prolyl isomerase Pin1. *Molecular and Cellular Biology*.

[63] Luisi BF, Xu WX, Otwinowski Z, Freedman LP, Yamamoto KR, Sigler PB (1991). Crystallographic analysis of the interaction of the glucocorticoid receptor with DNA. *Nature*.

[64] Schwabe JWR, Chapman L, Finch JT, Rhodes D (1993). The crystal structure of the estrogen receptor DNA-binding domain bound to DNA: how receptors discriminate between their response elements. *Cell*.

[65] Miyamoto T, Kakizawa T, Ichikawa K (2001). The role of hinge domain in heterodimerization and specific DNA recognition by nuclear receptors. *Molecular and Cellular Endocrinology*.

[66] Wurtz JM, Bourguet W, Renaud JP (1996). A canonical structure for the ligand-binding domain of nuclear receptors. *Nature Structural Biology*.

[67] Uppenberg J, Svensson C, Jaki M, Bertilsson G, Jendeberg L, Berkenstam A (1998). Crystal structure of the ligand binding domain of the human nuclear receptor PPAR*γ*. *Journal of Biological Chemistry*.

[68] Bourguet W, Ruff M, Chambon P, Gronemeyer H, Moras D (1995). Crystal structure of the ligand-binding domain of the human nuclear receptor RXR-*α*. *Nature*.

[69] Nayeri S, Kahlen JP, Carlberg G (1996). The high affinity ligand binding conformation of the nuclear 1,25-dihydroxyvitamin D3 receptor is functionally linked to the transactivation domain 2 (AF-2). *Nucleic Acids Research*.

[70] Brzozowski AM, Pike ACW, Dauter Z (1997). Molecular basis of agonism and antagonism in the oestrogen receptor. *Nature*.

[71] Wagner RL, Apriletti JW, McGrath ME, West BL, Baxter JD, Fletterick RJ (1995). A structural role for hormone in the thyroid hormone receptor. *Nature*.

[72] Huq MDM, Ha SG, Wei LN (2008). Modulation of retinoic acid receptor alpha activity by lysine methylation in the DNA binding domain. *Journal of Proteome Research*.

[73] La Rosa P, Pesiri V, Leclercq G, Marino M, Acconcia F (2012). Palmitoylation regulates 17*β*-estradiol-induced estrogen receptor *α* degradation and transcriptional activity. *Molecular Endocrinology*.

[74] Abdel-Hafiz HA, Horwitz KB (2012). Control of progesterone receptor ranscriptional synergy by SUMOylation and deSUMOylation. *BMC Molecular Biology*.

[75] Chen S, Gulla S, Cai C, Balk SP (2012). Androgen receptor serine 81 phosphorylation mediates chromatin binding and transcriptional activation. *Journal of Biological Chemistry*.

[76] Anbalagan M, Huderson B, Murphy L, Rowan BG (2012). Post-translational modifications of nuclear receptors and human disease. *Nuclear Receptor Signaling*.

[77] Wallace AD, Cidlowski JA (2001). Proteasome-mediated glucocorticoid receptor degradation restricts transcriptional signaling by glucocorticoids. *Journal of Biological Chemistry*.

[78] Ciechanover A, Orian A, Schwartz AL (2000). Ubiquitin-mediated proteolysis: biological regulation via destruction. *Bioessays*.

[79] Sengupta S, Wasylyk B (2001). Ligand-dependent interaction of the glucocorticoid receptor with p53 enhances their degradation by Hdm2. *Genes and Development*.

[80] Wang X, DeFranco DB (2005). Alternative effects of the ubiquitin-proteasome pathway on glucocorticoid receptor down-regulation and transactivation are mediated by CHIP, an E3 ligase. *Molecular Endocrinology*.

[81] Lonard DM, Nawaz Z, Smith CL, O’Malley BW (2000). The 26S proteasome is required for estrogen receptor-*α* and coactivator turnover and for efficient estrogen receptor-*α* transactivation. *Molecular Cell*.

[82] Deroo BJ, Rentsch C, Sampath S, Young J, DeFranco DB, Archer TK (2002). Proteasomal inhibition enhances glucocorticoid receptor transactivation and alters its subnuclear trafficking. *Molecular and Cellular Biology*.

[83] Pratt WB, Galigniana MD, Morishima Y, Murphy PJM (2004). Role of molecular chaperones in steroid receptor action. *Essays in Biochemistry*.

[84] Pratt WB, Morishima Y, Murphy M, Harrell M (2006). Chaperoning of glucocorticoid receptors. *Handbook of experimental pharmacology.*.

[85] Li L, Li Z, Howley PM, Sacks DB (2006). E6AP and calmodulin reciprocally regulate estrogen receptor stability. *Journal of Biological Chemistry*.

[86] Zhang Y, Li Z, Sacks DB, Ames JB (2012). Structural basis for Ca^2+^-induced activation and dimerization of estrogen receptor a by calmodulin. *Journal of Biological Chemistry*.

[87] Sivanandam A, Murthy S, Chinnakannu K (2011). Calmodulin protects androgen receptor from calpain-mediated breakdown in prostate cancer cells. *Journal of Cellular Physiology*.

[88] La Rosa P, Pesiri V, Leclercq G, Marino M, Acconcia F (2012). Palmitoylation regulates 17*β*-estradiol-induced estrogen receptor *α* degradation and transcriptional activity. *Molecular Endocrinology*.

[89] Chatterjee K, Lee JK, Rentoumis A, Jameson JL (1989). Negative regulation of the thyroid-stimulating hormone *α* gene by thyroid hormone: receptor interaction adjacent to the TATA box. *Proceedings of the National Academy of Sciences of the United States of America*.

[90] Seoane S, Alonso M, Segura C, Pérez-Fernández R (2002). Localization of a negative vitamin D response sequence in the human growth hormone gene. *Biochemical and Biophysical Research Communications*.

[91] Weisz A, Coppola L, Bresciani F (1986). Specific binding of estrogen receptor to sites upstream and within the transcribed region of the chicken ovalbumin gene. *Biochemical and Biophysical Research Communications*.

[92] La Baer J, Yamamoto KR (1994). Analysis of the DNA-binding affinity, sequence specificity and context dependence of the glucocorticoid receptor zinc finger region. *Journal of Molecular Biology*.

[93] Claessens F, Gewirth DT (2004). DNA recognition by nuclear receptors. *Essays in Biochemistry*.

[94] Tsai SY, Carlstedt-Duke J, Weigel NL (1988). Molecular interactions of steroid hormone receptor with its enhancer element: evidence for receptor dimer formation. *Cell*.

[95] Klinge CM, Bodenner DL, Desai D, Niles RM, Traish AM (1997). Binding of type II nuclear receptors and estrogen receptor to full and half-site estrogen response elements *in Vitro*. *Nucleic Acids Research*.

[96] Ribeiro RCJ, Feng W, Wagner RL (2001). Definition of the surface in the thyroid hormone receptor ligand binding domain for association as homodimers and heterodimers with retinoid X receptor. *Journal of Biological Chemistry*.

[97] Brelivet Y, Kammerer S, Rochel N, Poch O, Moras D (2004). Signature of the oligomeric behaviour of nuclear receptors at the sequence and structural level. *EMBO Reports*.

[98] Lee S, Privalsky ML (2005). Heterodimers of retinoic acid receptors and thyroid hormone receptors display unique combinatorial regulatory properties. *Molecular Endocrinology*.

[99] Powell E, Xu W (2008). Intermolecular interactions identify ligand-selective activity of estrogen receptor *α*/*β* dimers. *Proceedings of the National Academy of Sciences of the United States of America*.

[100] Wilson TE, Fahrner TJ, Milbrandt J (1993). The orphan receptors NGFI-B and steroidogenic factor 1 establish monomer binding as a third paradigm of nuclear receptor-DNA interaction. *Molecular and Cellular Biology*.

[101] Mangelsdorf DJ, Thummel C, Beato M (1995). The nuclear receptor super-family: the second decade. *Cell*.

[102] Danielsen M, Hinck L, Ringold GM (1989). Two amino acids within the knuckle of the first zinc finger specify DNA response element activation by the glucocorticoid receptor. *Cell*.

[103] Verrijdt G, Schoenmakers E, Haelens A (2000). Change of specificity mutations in androgen-selective enhancers. Evidence for a role of differential DNA binding by the androgen receptor. *Journal of Biological Chemistry*.

[104] Shaffer PL, Jivan A, Dollins DE, Claessens F, Gewirth DT (2004). Structural basis of androgen receptor binding to selective androgen response elements. *Proceedings of the National Academy of Sciences of the United States of America*.

[105] Denayer S, Helsen C, Thorrez L, Haelens A, Claessens F (2010). The rules of DNA recognition by the androgen receptor. *Molecular Endocrinology*.

[106] Aslam F, Shalhoub V, Van Wijnen AJ (1995). Contributions of distal and proximal promoter elements to glucocorticoid regulation of osteocalcin gene transcription. *Molecular Endocrinology*.

[107] Ou XM, Storring JM, Kushwaha N, Albert PR (2001). Heterodimerization of mineralocorticoid and glucocorticoid receptors at a novel negative response element of the 5-HT1A receptor gene. *Journal of Biological Chemistry*.

[108] Ruiz MMV, Bugge TH, Hirschmann P, Stunnenberg HG (1991). Functional characterization of a natural retinoic acid responsive element. *EMBO Journal*.

[109] Casas F, Daury L, Grandemange S (2003). Endocrine regulation of mitochondrial activity: involvement of truncated RXR*α* and c-Erb a*α*1 proteins. *FASEB Journal*.

[110] Williams GR, Zavacki AM, Harney JW, Brent GA (1994). Thyroid hormone receptor binds with unique properties to response elements that contain hexamer domains in an inverted palindrome arrangement. *Endocrinology*.

[111] Ikeda M, Rhee M, Chin WW (1994). Thyroid hormone receptor monomer, homodimer, and heterodimer (with retinoid-X receptor) contact different nucleotide sequences in thyroid hormone response elements. *Endocrinology*.

[112] Hirst MA, Hinck L, Danielsen M, Ringold GM (1992). Discrimination of DNA response elements for thyroid hormone and estrogen is dependent on dimerization of receptor DNA binding domains. *Proceedings of the National Academy of Sciences of the United States of America*.

[113] Towers TL, Luisi BF, Asianov A, Freedman LP (1993). DNA target selectivity by the vitamin D3 receptor: mechanism of dimer binding to an asymmetric repeat element. *Proceedings of the National Academy of Sciences of the United States of America*.

[114] Miyamoto T, Suzuki S, DeGroot LJ (1993). High affinity and specificity of dimeric binding of thyroid hormone receptors to DNA and their ligand-dependent dissociation. *Molecular Endocrinology*.

[115] Miyamoto T, Suzuki S, DeGroot LJ (1994). Differential binding and activation of thyroid hormone response elements by TR(*α*1) and RXR(*α*)-trap heterodimers. *Molecular and Cellular Endocrinology*.

[116] Carlberg C, Bendik I, Wyss A (1993). Two nuclear signalling pathways for vitamin D. *Nature*.

[117] Yan ZH, Medvedev A, Hirose T, Gotoh H, Jetten AM (1997). Characterization of the response element and DNA binding properties of the nuclear orphan receptor germ cell nuclear factor/retinoid receptor- related testis-associated receptor. *Journal of Biological Chemistry*.

[118] Schule R, Umesono K, Mangelsdorf DJ, Bolado J, Pike JW, Evans RM (1990). Jun-Fos and receptors for vitamins A and D recognize a common response element in the human osteocalcin gene. *Cell*.

[119] Lazar MA, Berrodin TJ (1990). Thyroid hormone receptors form distinct nuclear protein-dependent and independent complexes with a thyroid hormone response element. *Molecular Endocrinology*.

[120] Faisst S, Meyer S (1992). Compilation of vertebrate-encoded transcription factors. *Nucleic Acids Research*.

[121] Kirfel J, Kelter M, Cancela LM, Price PA, Schüle R (1997). Identification of a novel negative retinoic acid responsive element in the promoter of the human matrix Gla protein gene. *Proceedings of the National Academy of Sciences of the United States of America*.

[122] Eubank DW, Duplus E, Williams SC, Forest C, Beale EG (2001). Peroxisome proliferator-activated receptor *γ* and chicken ovalbumin upstream promoter transcription factor II negatively regulate the phosphoenolpyruvate carboxykinase promoter *via* a common element. *Journal of Biological Chemistry*.

[123] Kim MS, Fujiki R, Murayama A (2007). 1*β*,25(OH)_2_D_3_-induced transrepression by vitamin D receptor through E-box-type elements in the human parathyroid hormone gene promoter. *Molecular Endocrinology*.

[124] Guigon CJ, Kim DW, Zhu X, Zhao L, Cheng SY (2010). Tumor suppressor action of liganded thyroid hormone receptor *β* by direct repression of *β*-catenin gene expression. *Endocrinology*.

[125] Harding HP, Lazar MA (1993). The orphan receptor Rev-ErbA*α* activates transcription via a novel response element. *Molecular and Cellular Biology*.

[126] Giguere V, Tini M, Flock G, Ong E, Evans RM, Otulakowski G (1994). Isoform-specific amino-terminal domains dictate DNA-binding properties of ROR*α*, a novel family of orphan hormone nuclear receptors. *Genes and Development*.

[127] Pratt WB, Toft DO (1997). Steroid receptor interactions with heat shock protein and immunophilin chaperones. *Endocrine Reviews*.

[128] Graumann K, Jungbauer A (2000). Quantitative assessment of complex formation of nuclear-receptor accessory proteins. *Biochemical Journal*.

[129] Rajapandi T, Greene LE, Eisenberg E (2000). The molecular chaperones Hsp90 and Hsc70 are both necessary and sufficient to activate hormone binding by glucocorticoid receptor. *Journal of Biological Chemistry*.

[130] Cintron NS, Toft D (2006). Defining the requirements for Hsp40 and Hsp70 in the Hsp90 chaperone pathway. *Journal of Biological Chemistry*.

[131] Schülke JP, Wochnik GM, Lang-Rollin I (2010). Differential impact of tetratricopeptide repeat proteins on the steroid hormone receptors. *PloS one*.

[132] Nishi M, Kawata M (2007). Dynamics of glucocorticoid receptor and mineralocorticoid receptor: implications from live cell imaging studies. *Neuroendocrinology*.

[133] Kawata M, Nishi M, Matsuda K (2008). Steroid receptor signalling in the brain—lessons learned from molecular imaging. *Journal of Neuroendocrinology*.

[134] Elbi C, Walker DA, Romero G (2004). Molecular chaperones function as steroid receptor nuclear mobility factors. *Proceedings of the National Academy of Sciences of the United States of America*.

[135] Spencer TE, Jenster G, Burcin MM (1997). Steroid receptor coactivator-1 is a histone acetyltransferase. *Nature*.

[136] Liu Z, Wong J, Tsai SY, Tsai MJ, O’Malley BW (2001). Sequential recruitment of steroid receptor coactivator-1 (SRC-1) and p300 enhances progesterone receptor-dependent initiation and reinitiation of transcription from chromatin. *Proceedings of the National Academy of Sciences of the United States of America*.

[137] Kim MY, Hsiao SJ, Kraus WL (2001). A role for coactivators and histone acetylation in estrogen receptor *α*-mediated transcription initiation. *EMBO Journal*.

[138] Belandia B, Orford RL, Hurst HC, Parker MG (2002). Targeting of SWI/SNF chromatin remodelling complexes to estrogen-responsive genes. *EMBO Journal*.

[139] Kang Z, Jänne OA, Palvimo JJ (2004). Coregulator recruitment and histone modifications in transcriptional regulation by the androgen receptor. *Molecular Endocrinology*.

[140] van de Wijngaart DJ, Dubbink HJ, van Royen ME, Trapman J, Jenster G (2012). Androgen receptor coregulators: recruitment via the coactivator binding groove. *Molecular and Cellular Endocrinology*.

[141] Pearce D, Matsui W, Miner JN, Yamamoto KR (1998). Glucocorticoid receptor transcriptional activity determined by spacing of receptor and nonreceptor DNA sites. *Journal of Biological Chemistry*.

[142] Kushner PJ, Agard DA, Greene GL (2000). Estrogen receptor pathways to AP-1. *Journal of Steroid Biochemistry and Molecular Biology*.

[143] Takai H, Nakayama Y, Kim DS (2007). Androgen receptor stimulates bone sialoprotein (BSP) gene transcription via cAMP response element and activator protein 1/glucocorticoid response elements. *Journal of Cellular Biochemistry*.

[144] Konig H, Ponta H, Rahmsdorf HJ, Herrlich P (1992). Interference between pathway-specific transcription factors: glucocorticoids antagonize phorbol ester-induced AP-1 activity without altering AP-1 site occupation *in vivo*. *EMBO Journal*.

[145] Gionet N, Jansson D, Mader S, Pratt MAC (2009). NF-*κ*B and estrogen receptor *α* interactions: differential function in estrogen receptor-negative and -positive hormone-independent breast cancer cells. *Journal of Cellular Biochemistry*.

[146] Rao NA, McCalman MT, Moulos P (2011). Coactivation of GR and NFKB alters the repertoire of their binding sites and target genes. *Genome Research*.

[147] Chen JD, Evans RM (1995). A transcriptional co-repressor that interacts with nuclear hormone receptors. *Nature*.

[148] Hörlein AJ, Näär AM, Heinzel T (1995). Ligand-independent repression by the thyroid hormone receptor mediated by a nuclear receptor co-repressor. *Nature*.

[149] Cavailles V, Dauvois S, L’Horset F (1995). Nuclear factor RIP140 modulates transcriptional activation by the estrogen receptor. *EMBO Journal*.

[150] Fernandes I, Bastien Y, Wai T (2003). Ligand-dependent nuclear receptor corepressor LCoR functions by histone deacetylase-dependent and -independent mechanisms. *Molecular Cell*.

[151] Augereau P, Badia E, Carascossa S (2006). The nuclear receptor transcriptional coregulator RIP140. *Nuclear Receptor Signaling*.

[152] Subramaniam N, Cairns W, Okret S (1998). Glucocorticoids repress transcription from a negative glucocorticoid response element recognized by two homeodomain-containing proteins, Pbx and Oct-1. *Journal of Biological Chemistry*.

[153] Wilson MA, Chrysogelos SA (2002). Identification and characterization of a negative regulatory element within the epidermal growth factor receptor gene first intron in hormone-dependent breast cancer cells. *Journal of Cellular Biochemistry*.

[154] Govindan MV (2010). Recruitment of cAMP-response element-binding protein and histone deacetylase has opposite effects on glucocorticoid receptor gene transcription. *Journal of Biological Chemistry*.

[155] Prüfer K, Barsony J (2002). Retinoid X Receptor dominates the nuclear import and export of the unliganded vitamin D receptor. *Molecular Endocrinology*.

[156] Cao X, Liu W, Lin F (2004). Retinoid X receptor regulates Nur77/TR3-dependent apoptosis by modulating its nuclear export and mitochondrial targeting. *Molecular and Cellular Biology*.

[157] Yu VC, Delsert C, Andersen B (1991). RXR*β*: a coregulator that enhances binding of retinoic acid, thyroid hormone, and vitamin D receptors to their cognate response elements. *Cell*.

[158] Puzianowska-Kuznicka M, Damjanovski S, Shi YB (1997). Both thyroid hormone and 9-*cis* retinoic acid receptors are required to efficiently mediate the effects of thyroid hormone on embryonic development and specific gene regulation in *Xenopus* laevis. *Molecular and Cellular Biology*.

[159] Laflamme L, Hamann G, Messier N, Maltais S, Langlois MF (2002). RXR acts as a coregulator in the regulation of genes of the hypothalamo-pituitary axis by thyroid hormone receptors. *Journal of Molecular Endocrinology*.

[160] Lefebvre P, Benomar Y, Staels B (2010). Retinoid X receptors: common heterodimerization partners with distinct functions. *Trends in Endocrinology and Metabolism*.

[161] Mader S, Chen JY, Chen Z, White J, Chambon P, Gronemeyer H (1993). The patterns of binding of RAR, RXR and TR homo- and heterodimers to direct repeats are dictated by the binding specificities of the DNA binding domains. *EMBO Journal*.

[162] Force WR, Tillman JB, Sprung CN, Spindler SR (1994). Homodimer and heterodimer DNA binding and transcriptional responsiveness to triiodothyronine (T3) and 9-cis-retinoic acid are determined by the number and order of high affinity half-sites in a T3 response element. *Journal of Biological Chemistry*.

[163] Morin B, Nichols LA, Holland LJ (2006). Flanking sequence composition differentially affects the binding and functional characteristics of glucocorticoid receptor homo- and heterodimers. *Biochemistry*.

[164] Perlmann T, Rangarajan PN, Umesono K, Evans RM (1993). Determinants for selective RAR and TR recognition of direct repeat HREs. *Genes and Development*.

[165] Kurokawa R, Yu VC, Naar A (1993). Differential orientations of the DNA-binding domain and carboxy-terminal dimerization interface regulate binding site selection by nuclear receptor heterodimers. *Genes and Development B*.

[166] Kurokawa R, DiRenzo J, Boehm M (1994). Regulation of retinoid signalling by receptor polarity and allosteric control of ligand binding. *Nature*.

[167] IJpenberg A, Jeannin E, Wahli W, Desvergne B (1997). Polarity and specific sequence requirements of peroxisome proliferator- activated receptor (PPAR)/retinoid X receptor heterodimer binding to DNA. A functional analysis of the malic enzyme gene PPAR response element. *Journal of Biological Chemistry*.

[168] Ferrara J, McCuaig K, Hendy GN, Uskokovic M, White JH (1994). Highly potent transcriptional activation by 16-ene derivatives of 1,25- dihydroxyvitamin D3. Lack of modulation by 9-*cis*-retinoic acid of response to 1,25-dihydroxyvitamin D3 or its derivatives. *Journal of Biological Chemistry*.

[169] Forman BM, Umesono K, Chen J, Evans RM (1995). Unique response pathways are established by allosteric interactions among nuclear hormone receptors. *Cell*.

[170] Li D, Yamada T, Wang F, Vulin AI, Samuels HH (2004). Novel roles of retinoid X receptor (RXR) anal RXR ligand in dynamically modulating the activity of the thyroid hormone receptor/RXR heterodimer. *Journal of Biological Chemistry*.

[171] Chen JY, Clifford J, Zusi C (1996). Two distinct actions of retinoid-receptor ligands. *Nature*.

[172] Kliewer SA, Umesono K, Noonan DJ, Heyman RA, Evans RM (1992). Convergence of 9-cis retinoic acid and peroxisome proliferator signalling pathways through heterodimer formation of their receptors. *Nature*.

[173] Antonio V, Janvier B, Brouillet A, Andreani M, Raymondjean M (2003). Oxysterol and 9-cis-retinoic acid stimulate the group IIA secretory phospholipase A2 gene in rat smooth-muscle cells. *Biochemical Journal*.

[174] Zamir I, Dawson J, Lavinsky RM, Glass CK, Rosenfeld MG, Lazar MA (1997). Cloning and characterization of a corepressor and potential component of the nuclear hormone receptor repression complex. *Proceedings of the National Academy of Sciences of the United States of America*.

[175] Dressel U, Thormeyer D, Altincicek B (1999). Alien, a highly conserved protein with characteristics of a corepressor for members of the nuclear hormone receptor superfamily. *Molecular and Cellular Biology*.

[176] Watson PJ, Fairall L, Schwabe JW (2012). Nuclear hormone receptor co-repressors: structure and function. *Molecular and Cellular Endocrinology*.

[177] Kwok RPS, Lundblad JR, Chrivia JC (1994). Nuclear protein CBP is a coactivator for the transcription factor CREB. *Nature*.

[178] Lundblad JR, Kwok RPS, Laurance ME, Harter ML, Goodman RH (1995). Adenoviral E1A-associated protein p300 as a functional homologue of the transcriptional co-activator CBP. *Nature*.

[179] Onate SA, Tsai SY, Tsai MJ, O’Malley BW (1995). Sequence and characterization of a coactivator for the steroid hormone receptor superfamily. *Science*.

[180] Voegel JJ, Heine MJS, Zechel C, Chambon P, Gronemeyer H (1996). TIF2, a 160 kDa transcriptional mediator for the ligand-dependent activation function AF-2 of nuclear receptors. *EMBO Journal*.

[181] Fondell JD, Ge H, Roeder RG (1996). Ligand induction of a transcriptionally active thyroid hormone receptor coactivator complex. *Proceedings of the National Academy of Sciences of the United States of America*.

[182] Yang XJ, Ogryzko VV, Nishikawa JI, Howard BH, Nakatani Y (1996). A p300/CPB-associated factor that competes with the adenoviral oncoprotein E1A. *Nature*.

[183] Takeshita A, Cardona GR, Koibuchi N, Suen CS, Chin WW (1997). TRAM-1, a novel 160-kDa thyroid hormone receptor activator molecule, exhibits distinct properties from steroid receptor coactivator-1. *Journal of Biological Chemistry*.

[184] Puigserver P, Wu Z, Park CW, Graves R, Wright M, Spiegelman BM (1998). A cold-inducible coactivator of nuclear receptors linked to adaptive thermogenesis. *Cell*.

[185] Rachez C, Suldan Z, Ward J (1998). A novel protein complex that interacts with the vitamin D3 receptor in a ligand-dependent manner and enhances VDR transactivation in a cell-free system. *Genes and Development*.

[186] Kim HJ, Yi JY, Sung HS (1999). Activating signal cointegrator 1, a novel transcription coactivator of nuclear receptors, and its cytosolic localization under conditions of serum deprivation. *Molecular and Cellular Biology*.

[187] Lee SK, Anzick SL, Choi JE (1999). A nuclear factor, ASC-2, as a cancer-amplified transcriptional coactivator essential for ligand-dependent transactivation by nuclear receptors *in vivo*. *Journal of Biological Chemistry*.

[188] Wu Y, Delerive P, Chin WW, Burris TP (2002). Requirement of helix 1 and the AF-2 domain of the thyroid hormone receptor for coactivation by PGC-1. *Journal of Biological Chemistry*.

[189] York B, O’Malley BW (2010). Steroid Receptor Coactivator (SRC) family: masters of systems biology. *Journal of Biological Chemistry*.

[190] Salbert G, Fanjul A, Piedrafita FJ (1993). Retinoic acid receptors and retinoid X receptor-*α* down-regulate the transforming growth factor-*β*1 promoter by antagonizing AP-1 activity. *Molecular Endocrinology*.

[191] Harant H, Andrew PJ, Reddy GS, Foglar E, Lindley IJD (1997). 1*α*,25-dihydroxyvitamin D3 and a variety of its natural metabolites transcriptionally repress nuclear-factor-*κ*B-mediated interleukin-8 gene expression. *European Journal of Biochemistry*.

[192] Barrera-Hernandez G, Zhan Q, Wong R, Cheng SY (1998). Thyroid hormone receptor is a negative regulator in p53-mediated signaling pathways. *DNA and Cell Biology*.

[193] Xu J, Thompson KL, Shephard LB, Hudson LG, Gill GN (1993). T3 receptor suppression of Sp1-dependent transcription from the epidermal growth factor receptor promoter via overlapping DNA-binding sites. *Journal of Biological Chemistry*.

[194] D’Ambrosio D, Cippitelli M, Cocciolo MG (1998). Inhibition of IL-12 production by 1,25-dihydroxyvitamin D3. Involvement of NF-*κ*B downregulation in transcriptional repression of the p40 gene. *Journal of Clinical Investigation*.

[195] Potter GB, Beaudoin GMJ, DeRenzo CL, Zarach JM, Chen SH, Thompson CC (2001). The hairless gene mutated in congenital hair loss disorders encodes a novel nuclear receptor corepressor. *Genes and Development*.

[196] Moraitis AN, Giguère V, Thompson CC (2002). Novel mechanism of nuclear receptor corepressor interaction dictated by activation function 2 helix determinants. *Molecular and Cellular Biology*.

[197] Hsieh JC, Sisk JM, Jurutka PW (2003). Physical and functional interaction between the vitamin D receptor and hairless corepressor, two proteins required for hair cycling. *Journal of Biological Chemistry*.

[198] Epping MT, Wang L, Edel MJ, Carlée L, Hernandez M, Bernards R (2005). The human tumor antigen PRAME is a dominant repressor of retinoic acid receptor signaling. *Cell*.

[199] Hashimoto K, Yamada M, Matsumoto S, Monden T, Satoh T, Mori M (2006). Mouse sterol response element binding protein-1c gene expression is negatively regulated by thyroid hormone. *Endocrinology*.

[200] Blanco JCG, Wang IM, Tsai SY (1995). Transcription factor TFIIB and the vitamin D receptor cooperatively activate ligand-dependent transcription. *Proceedings of the National Academy of Sciences of the United States of America*.

[201] Fondell JD, Brunel F, Hisatake K, Roeder RG (1996). Unliganded thyroid hormone receptor *α* can target TATA-binding protein for transcriptional repression. *Molecular and Cellular Biology*.

[202] Mcewan IJ, Gustafsson J (1997). Interaction of the human androgen receptor transactivation function with the general transcription factor TFIIF. *Proceedings of the National Academy of Sciences of the United States of America*.

[203] Wärnmark A, Wikström A, Wright APH, Gustafsson JÅ, Härd T (2001). The N-terminal Regions of Estrogen Receptor *α* and *β* are Unstructured *in Vitro* and Show Different TBP Binding Properties. *Journal of Biological Chemistry*.

[204] Reid J, Murray I, Watt K, Betney R, McEwan IJ (2002). The androgen receptor interacts with multiple regions of the large subunit of general transcription factor TFIIF. *Journal of Biological Chemistry*.

[205] Khan SH, Ling J, Kumar R (2011). TBP binding-induced folding of the Glucocorticoid receptor AF1 domain facilitates its interaction with Steroid Receptor Coactivator-1. *PLoS ONE*.

[206] Heery DM, Kalkhoven E, Hoare S, Parker MG (1997). A signature motif in transcriptional co-activators mediates binding to nuclear receptors. *Nature*.

[207] Bannister AJ, Kouzarides T (1996). The CBP co-activator is a histone acetyltransferase. *Nature*.

[208] Ogryzko VV, Schiltz RL, Russanova V, Howard BH, Nakatani Y (1996). The transcriptional coactivators p300 and CBP are histone acetyltransferases. *Cell*.

[209] Shao W, Rosenauer A, Mann K (2000). Ligand-inducible interaction of the DRIP/TRAP coactivator complex with retinoid receptors in retinoic acid-sensitive and -resistant acute promyelocytic leukemia cells. *Blood*.

[210] Perissi V, Staszewski LM, McInerney EM (1999). Molecular determinants of nuclear receptor-corepressor interaction. *Genes and Development*.

[211] Webb P, Anderson CM, Valentine C (2000). The nuclear receptor corepressor (N-CoR) contains three isoleucine motifs (I/LXXII) that serve as receptor interaction domains (IDs). *Molecular Endocrinology*.

[212] Guenther MG, Barak O, Lazar MA (2001). The SMRT and N-CoR corepressors are activating cofactors for histone deacetylase 3. *Molecular and Cellular Biology*.

[213] Codina A, Love JD, Li Y, Lazar MA, Neuhaus D, Schwabe JWR (2005). Structural insights into the interaction and activation of histone deacetylase 3 by nuclear receptor corepressors. *Proceedings of the National Academy of Sciences of the United States of America*.

[214] Montano MM, Ekena K, Delage-Mourroux R, Chang W, Martini P, Katzenellenbogen BS (1999). An estrogen receptor-selective coregulator that potentiates the effectiveness of antiestrogens and represses the activity of estrogens. *Proceedings of the National Academy of Sciences of the United States of America*.

[215] Mazumdar A, Wang RA, Mishra SK (2001). Transcriptional repression of oestrogen receptor by metastasis-associated protein 1 corepressor. *Nature Cell Biology*.

[216] Huang N, Vom Baur E, Garnier JM (1998). Two distinct nuclear receptor interaction domains in NSD1, a novel SET protein that exhibits characteristics of both corepressors and coactivators. *EMBO Journal*.

[217] Scheinman RI, Gualberto A, Jewell CM, Cidlowski JA, Baldwin AS (1995). Characterization of mechanisms involved in transrepression of NF-*κ*B by activated glucocorticoid receptors. *Molecular and Cellular Biology*.

[218] Yap N, Yu CL, Cheng SY (1996). Modulation of the transcriptional activity of thyroid hormone receptors by the tumor suppressor p53. *Proceedings of the National Academy of Sciences of the United States of America*.

[219] Préfontaine GG, Walther R, Giffin W, Lemieux ME, Pope L, Haché RJG (1999). Selective binding of steroid hormone receptors to octamer transcription factors determines transcriptional synergism at the mouse mammary tumor virus promoter. *Journal of Biological Chemistry*.

[220] Delerive P, De Bosscher K, Besnard S (1999). Peroxisome proliferator-activated receptor *α* negatively regulates the vascular inflammatory gene response by negative cross-talk with transcription factors NF-*κ*B and AP-1. *Journal of Biological Chemistry*.

[221] Wang LH, Yang XY, Zhang X (2004). Transcriptional inactivation of STAT3 by PPARg suppresses IL-6-responsive multiple myeloma cells. *Immunity*.

[222] Kim HP, Lee JY, Jeong JK, Bae SW, Lee HK, Jo I (1999). Nongenomic stimulation of nitric oxide release by estrogen is mediated by estrogen receptor *α* localized in caveolae. *Biochemical and Biophysical Research Communications*.

[223] Lu ML, Schneider MC, Zheng Y, Zhang X, Richie JP (2001). Caveolin-1 interacts with androgen receptor. A positive modulator of androgen receptor mediated transactivation. *Journal of Biological Chemistry*.

[224] Huhtakangas JA, Olivera CJ, Bishop JE, Zanello LP, Norman AW (2004). The vitamin D receptor is present in caveolae-enriched plasma membranes and binds 1*α*,25(OH)_2_-vitamin D_3_
*in vivo* and *in Vitro*. *Molecular Endocrinology*.

[225] Matthews L, Berry A, Ohanian V, Ohanian J, Garside H, Ray D (2008). Caveolin mediates rapid glucocorticoid effects and couples glucocorticoid action to the antiproliferative program. *Molecular Endocrinology*.

[226] Mousa SA, O’Connor LJ, Bergh JJ, Davis FB, Scanlan TS, Davis PJ (2005). The proangiogenic action of thyroid hormone analogue GC-1 is initiated at an integrin. *Journal of Cardiovascular Pharmacology*.

[227] Karteris E, Zervou S, Pang Y (2006). Progesterone signaling in human myometrium through two novel membrane G protein-coupled receptors: potential role in functional progesterone withdrawal at term. *Molecular Endocrinology*.

[228] Smith JL, Kupchak BR, Garitaonandia I (2008). Heterologous expression of human mPR*α*, mPR*β* and mPR*γ* in yeast confirms their ability to function as membrane progesterone receptors. *Steroids*.

[229] Nakhla AM, Khan MS, Rosner W (1990). Biologically active steroids activate receptor-bound human sex hormone-binding globulin to cause LNCaP cells to accumulate adenosine 3′,5′-monophosphate. *Journal of Clinical Endocrinology and Metabolism*.

[230] Nakhla AM, Leonard J, Hryb DJ, Rosner W (1999). Sex hormone-binding globulin receptor signal transduction proceeds via a G protein. *Steroids*.

[231] Mitchell EA, Herd MB, Gunn BG, Lambert JJ, Belelli D (2008). Neurosteroid modulation of GABAA receptors: molecular determinants and significance in health and disease. *Neurochemistry International*.

[232] Acconcia F, Ascenzi P, Bocedi A (2005). Palmitoylation-dependent estrogen receptor *α* membrane localization: regulation by 17*β*-estradiol. *Molecular Biology of the Cell*.

[233] Li L, Haynes MP, Bender JR (2003). Plasma membrane localization and function of the estrogen receptor *α* variant (ER46) in human endothelial cells. *Proceedings of the National Academy of Sciences of the United States of America*.

[234] Figtree GA, McDonald D, Watkins H, Channon KM (2003). Truncated estrogen receptor *α* 46-kDa isoform in human endothelial cells: relationship to acute activation of nitric oxide synthase. *Circulation*.

[235] Wang Z, Zhang X, Shen P, Loggie BW, Chang Y, Deuel TF (2006). A variant of estrogen receptor-*α*, hER-*α*36: transduction of estrogen- and antiestrogen-dependent membrane-initiated mitogenic signaling. *Proceedings of the National Academy of Sciences of the United States of America*.

[236] Razandi M, Pedram A, Levin ER (2010). Heat shock protein 27 is required for sex steroid receptor trafficking to and functioning at the plasma membrane. *Molecular and Cellular Biology*.

[237] Pedram A, Razandi M, Deschenes RJ, Levin ER (2012). DHHC-7 and -21 are palmitoylacyltransferases for sex steroid receptors. *Molecular Biology of the Cell*.

[238] Pedram A, Razandi M, Sainson RCA, Kim JK, Hughes CC, Levin ER (2007). A conserved mechanism for steroid receptor translocation to the plasma membrane. *Journal of Biological Chemistry*.

[239] Bondar G, Kuo J, Hamid N, Micevych P (2009). Estradiol-induced estrogen receptor-*α* trafficking. *Journal of Neuroscience*.

[240] Buitrago C, Boland R (2010). Caveolae and caveolin-1 are implicated in 1*α*,25(OH)2-vitamin D3-dependent modulation of Src, MAPK cascades and VDR localization in skeletal muscle cells. *Journal of Steroid Biochemistry and Molecular Biology*.

[241] Bennett N, Hooper JD, Lee CS, Gobe GC (2009). Androgen receptor and caveolin-1 in prostate cancer. *IUBMB Life*.

[242] Zhao G, Simpson RU (2010). Membrane localization, Caveolin-3 association and rapid actions of vitamin D receptor in cardiac myocytes. *Steroids*.

[243] Schwartz Z, Ehland H, Sylvia VL (2002). 1*α*,25-dihydroxyvitamin D3 and 24R,25-dihydroxyvitamin D3 modulate growth plate chondrocyte physiology via protein kinase C-dependent phosphorylation of extracellular signal-regulated kinase 1/2 mitogen-activated protein kinase. *Endocrinology*.

[244] Vicencio JM, Ibarra C, Estrada M (2006). Testosterone induces an intracellular calcium increase by a nongenomic mechanism in cultured rat cardiac myocytes. *Endocrinology*.

[245] Storey NM, Gentile S, Ullah H (2006). Rapid signaling at the plasma membrane by a nuclear receptor for thyroid hormone. *Proceedings of the National Academy of Sciences of the United States of America*.

[246] Zamoner A, Heimfarth L, Loureiro SO, Royer C, Silva FRMB, Pessoa-Pureur R (2008). Nongenomic actions of thyroxine modulate intermediate filament phosphorylation in cerebral cortex of rats. *Neuroscience*.

[247] Elbaradie K, Wang Y, Boyan BD, Schwartz Z (2012). Rapid membrane responses to dihydrotestosterone are sex dependent in growth plate chondrocytes. *Journal of Steroid Biochemistry and Molecular Biology*.

[248] Menegaz D, Barrientos-Duran A, Kline A (2010). 1*α*,25(OH)_2_-Vitamin D_3_ stimulation of secretion via chloride channel activation in Sertoli cells. *Journal of Steroid Biochemistry and Molecular Biology*.

[249] Druzin M, Malinina E, Grimsholm O, Johansson S (2011). Mechanism of estradiol-induced block of voltage-gated K^+^ currents in rat medial preoptic neurons. *PLoS ONE*.

[250] Simoncini T, Hafezi-Moghadam A, Brazil DP, Ley K, Chin WW, Llao JK (2000). Interaction of oestrogen receptor with the regulatory subunit of phosphatidylinositol-3-OH kinase. *Nature*.

[251] Pietrzak M, Puzianowska-Kuznicka M (2008). Triiodothyronine utilizes phosphatidylinositol 3-kinase pathway to activate anti-apoptotic myeloid cell leukemia-1. *Journal of Molecular Endocrinology*.

[252] Yan TD, Wu H, Zhang HP (2010). Oncogenic potential of retinoic acid receptor g in hepatocellular carcinoma. *Cancer Research*.

[253] Boonyaratanakornkit V, Scott MP, Ribon V (2001). Progesterone receptor contains a proline-rich motif that directly interacts with SH3 domains and activates c-Src family tyrosine kinases. *Molecular Cell*.

[254] Migliaccio A, Varricchio L, De Falco A (2007). Inhibition of the SH3 domain-mediated binding of Src to the androgen receptor and its effect on tumor growth. *Oncogene*.

[255] Cheskis BJ, Greger J, Cooch N (2008). MNAR plays an important role in ERa activation of Src/MAPK and PI3K/Akt signaling pathways. *Steroids*.

[256] García-Pedrero JM, Rio BD, Martínez-Campa C, Muramatsu M, Lazo PS, Ramos S (2002). Calmodulin is a selective modulator of estrogen receptors. *Molecular Endocrinology*.

[257] Li L, Li Z, Sacks DB (2005). The transcriptional activity of estrogen receptor-*α* is dependent on Ca^2+^/calmodulin. *Journal of Biological Chemistry*.

[258] Hentschke M, Schulze C, Süsens U, Borgmeyer U (2003). Characterization of calmodulin binding to the orphan nuclear receptor ERR*γ*. *Biological Chemistry*.

[259] Cifuentes E, Mataraza JM, Yoshida BA (2004). Physical and functional interaction of androgen receptor with calmodulin in prostate cancer cells. *Proceedings of the National Academy of Sciences of the United States of America*.

[260] Alzamora R, Brown LR, Harvey BJ (2007). Direct binding and activation of protein kinase C isoforms by aldosterone and 17*β*-estradiol. *Molecular Endocrinology*.

[261] Alzamora R, Harvey BJ (2008). Direct binding and activation of protein kinase C isoforms by steroid hormones. *Steroids*.

[262] Demonacos C, Tsawdaroglou NC, Djordjevic-Markovic R (1993). Import of the glucocorticoid receptor into rat liver mitochondria *in vivo* and *in Vitro*. *Journal of Steroid Biochemistry and Molecular Biology*.

[263] Wrutniak C, Cassar-Malek I, Marchal S (1995). A 43-kDa protein related to c-Erb A *α*1 is located in the mitochondrial matrix of rat liver. *Journal of Biological Chemistry*.

[264] Casas F, Domenjoud L, Rochard P (2000). A 45 kDa protein related to PPAR*γ*2, induced by peroxisome proliferators, is located in the mitochondrial matrix. *FEBS Letters*.

[265] Chen JQ, Eshete M, Alworth WL, Yager JD (2004). Binding of MCF-7 cell mitochondrial proteins and recombinant human estrogen receptors *α* and *β* to human mitochondrial DNA estrogen response elements. *Journal of Cellular Biochemistry*.

[266] Arnold S, Goglia F, Kadenbach B (1998). 3,5-Diiodothyronine binds to subunit Va of cytochrome-c oxidase and abolishes the allosteric inhibition of respiration by ATP. *European Journal of Biochemistry*.

[267] Notario B, Zamora M, Viñas O, Mampel T (2003). All-*trans*-retinoic acid binds to and inhibits adenine nucleotide translocase and induces mitochondrial permeability transition. *Molecular Pharmacology*.

[268] Sterling K, Brenner MA (1995). Thyroid hormone action: effect of triiodothyronine on mitochondrial adenine nucleotide translocase *in vivo* and *in Vitro*. *Metabolism*.

[269] Lin B, Kolluri SK, Lin F (2004). Conversion of Bcl-2 from protector to killer by interaction with nuclear orphan receptor Nur77/TR3. *Cell*.

[270] Horvat A, Petrović S, Nedeljković N, Martinović JV, Nikezić G (2000). Estradiol affect Na-dependent Ca^2+^ efflux from synaptosomal mitochondria. *General Physiology and Biophysics*.

[271] Saelim N, John LM, Wu J (2004). Nontranscriptional modulation of intracellular Ca^2+^ siqnaling by ligand stimulated thyroid hormone receptor. *Journal of Cell Biology*.

[272] Zhang L, Zhou R, Li X, Ursano RJ, Li H (2006). Stress-induced change of mitochondria membrane potential regulated by genomic and non-genomic GR signaling: a possible mechanism for hippocampus atrophy in PTSD. *Medical Hypotheses*.

[273] Poon MM, Chen L (2008). Retinoic acid-gated sequence-specific translational control by RAR*α*. *Proceedings of the National Academy of Sciences of the United States of America*.

[274] Buttgereit F, Scheffold A (2002). Rapid glucocorticoid effects on immune cells. *Steroids*.

[275] Panin LE, Mokrushnikov PV, Kunitsyn VG, Zaitsev BN (2010). Interaction mechanism of cortisol and catecholamines with structural components of erythrocyte membranes. *Journal of Physical Chemistry B*.

